# Identification of small cell lung cancer patients who are at risk of developing common serious adverse event groups with machine learning

**DOI:** 10.3389/fdsfr.2023.1267623

**Published:** 2023-09-15

**Authors:** Linda Wanika, Neil D. Evans, Michael J. Chappell

**Affiliations:** School of Engineering, University of Warwick, Coventry, United Kingdom

**Keywords:** small cell lung cancer, serious adverse events, grade classification, chemotherapy, machine learning

## Abstract

**Introduction:** Across multiple studies, the most common serious adverse event groups that Small Cell Lung Cancer (SCLC) patients experience, whilst undergoing chemotherapy treatment, are: Blood and Lymphatic Disorders, Infections and Infestations together with Metabolism and Nutrition Disorders. The majority of the research that investigates the relationship between adverse events and SCLC patients, focuses on specific adverse events such as neutropenia and thrombocytopenia.

**Aim:** This study aims to utilise machine learning in order to identify those patients who are at risk of developing common serious adverse event groups, as well as their specific adverse event classification grade.

**Methods:** Data from five clinical trial studies were analysed and 12 analysis groups were formed based on the serious adverse event group and grade.

**Results:** The best test runs for each of the models were able to produce an area under the curve (AUC) score of at least 0.714. The best model was the Blood and Lymphatic Disorder group, SAE grade 0 vs. grade 3 (best AUC = 1, sensitivity rate = 0.84, specificity rate = 0.96).

**Conclusion:** The top features that contributed to this prediction were total bilirubin, alkaline phosphatase, and age. Future work should investigate the relationship between these features and common SAE groups.

## 1 Introduction

Lung cancer is one of the most common cancers worldwide. Approximately 7.5% of people are at risk of developing lung cancer (Cancer Research UK, 2023; World Cancer Research Fund International, 2023). Small Cell Lung Cancer (SCLC) accounts for 15% of lung cancer cases (Kahnert et al., 2016). While the majority of lung cancer diagnoses are Non-Small Cell Lung Cancer cases, SCLC patients in general have a higher metastasise rate (Krohn et al., 2014). Treatment options for SCLC aim to simply manage the disease (Deneka et al., 2019). Chemotherapy is one of the main treatment options for SCLC with the goal of reducing the spread of the tumour through the disruption of the tumour DNA replication and cell division process (Kitao et al., 2017). Common examples of such chemotherapy include carboplatin and cisplatin (Oun et al., 2018; Azab et al., 2019).

As with all medications, patients may experience adverse events whilst undergoing chemotherapy treatment. Adverse events are unintended effects in response to a treatment therapy. Adverse events can be classified into different grades according to the common terminology criteria for adverse events (CTCAE) (Trotti et al., 2003; Cancer Therapy Evaluation Program, 2023). Adverse events that are grades 1 or 2 tend to be relatively mild to moderate and often do not require serious medical intervention. In the context of this paper, serious adverse events (SAEs) are adverse events that are classed as grade 3 or higher. For grade 3 events, patients may require hospitalisation and their quality of life may begin to be severely affected. In grade 4, the SAE is considered to be life threatening and the patient is in need of urgent medical care. SAE grade 5 is death caused by an adverse event (Cancer Therapy Evaluation Program, 2023). SAEs remain a critical challenge as many patients who experience SAEs, may be suspended from their medical treatment. This unfortunately increases the likelihood that their tumour may begin to thrive, proliferate and has the potential to metastasise. There are also potential ongoing costs for other medications that need to be used. Some SAEs also have no defined mechanism which is a challenge in terms of being able to predict which patients are at risk (Duncan et al., 2015). There is a need to identify which patients are likely to develop SAEs as this can aid in specialised monitoring of at-risk patients while they commence and maintain their prescribed treatment.

One of the most commonly experienced SAEs in patients who develop SCLC is neutropenia. This SAE belongs to the Blood and Lymphatic system Disorders adverse event group (Kishida et al., 2009; Cancer Therapy Evaluation Program, 2023). Neutropenia is a term used to describe low neutrophil levels. Patients with neutropenia are generally more susceptible to infections and sepsis (Nesher and Rolston, 2013; Kochanek et al., 2019). There are many studies which have identified risk factors for neutropenia through the use of machine learning (Cho et al., 2020; Venäläinen et al., 2021; Wiberg et al., 2021). Machine learning uses algorithms in order to uncover possible relationships between variables in a dataset. Supervised classification machine learning can be used to assess the relationship between different input features in order to predict a particular response, such as whether or not a patient may experience neutropenia (Nasteski, 2017). Example risk factors for neutropenia include age and low blood cell count (Lyman et al., 2014).

While the vast majority of SAEs experienced by SCLC patients who are treated with chemotherapy agents is neutropenia, there are other SAEs from different adverse event groups that patients may also be at risk of developing (Ludwig et al., 2014; McQuade et al., 2020). Moreover, many of the machine learning studies that are published for adverse events in general focus more on the comparison between patients who do not experience an adverse event (the control group) and those patients who do indeed develop the adverse event. Many machine learning classification algorithms are primarily used to determine two possible outcomes. However, SAEs can potentially have 4 different classifications, no SAE or grade 0, SAE grade 3, SAE grade 4 and SAE grade 5. Note, that there is no SAE grade 1 or 2 in order to avoid confusion with adverse events grade 1 and 2 which are not SAEs. The identification of not only which SAE group a patient is likely to be a member of, but also the grade, before a patient has commenced their chemotherapy treatment, would offer many benefits. For example, the patients who are identified as being at risk of developing a particular SAE group may receive closer monitoring and they could potentially be prescribed other medications to help combat the potential onset of a particular SAE group. For patients who are identified as at risk of developing a SAE grade 5, these patients may be given an alternative cancer treatment or have their dosages reduced, in order to mitigate the risk of more serious consequences, including death, due to the SAE.

The aims of this investigation are to identify those patients who are at risk of developing SAEs from commonly occurring SAE groups, as well as their classifications. Moreover, this study aims to highlight any predictive features that may make a patient susceptible to developing a particular SAE group.

## 2 Materials and methods

### 2.1 Clinical data collection

Access to SCLC clinical trial data was obtained through Project Data Sphere (Project Data Sphere, 2023). Data from the following five clinical trial studies were used: NCT02499770, NCT00143455, NCT01439568, NCT00119613 and NCT00363415 (ClinicalTrials.gov:NCT02499770, 2020; ClinicalTrials.gov:NCT00143455, 2010; ClinicalTrials.gov:NCT01439568, 2019; ClinicalTrials.gov:NCT00119613, 2008; ClinicalTrials.gov:NCT00363415, 2009). These studies were selected based on the accessibility of the data and the inclusion of laboratory data. All of these studies had patients who were assigned to start a chemotherapy treatment. The data from these studies were merged together in order to analyse the occurrence of SAEs in SCLC patients. A total of 1,043 patients were eligible for the analysis. Table 1 provides a summary of the baseline characteristics that were included in the analysis.

**TABLE 1:** Summary baseline features that were included in the analysis.

Features	Total N: 1,043	No SAE N: 289	Yes SAE N: 754
Demographic			
Age: below 45 Yrs (count (%))	26 (2.5)	6 (2.1)	20 (2.7)
Age: 45 to 49 Yrs (count (%))	59 (5.7)	28 (9.7)	31 (4.1)
Age: 50 to 54 Yrs (count (%))	147 (14.1)	49 (17)	98 (13)
Age: 55 to 59 Yrs (count (%))	193 (18.5)	58 (20.1)	135 (17.9)
Age: 60 to 64 Yrs (count (%))	217 (20.8)	55 (19)	162 (21.5)
Age: 65 to 69 Yrs (count (%))	205 (19.7)	54 (18.7)	151 (20)
Age: 70 to 74 Yrs (count (%))	122 (11.7)	26 (9)	96 (12.7)
Age: 75 to 79 Yrs (count (%))	56 (5.4)	9 (3.1)	47 (6.2)
Age: 80 or above Yrs (count (%))	18 (1.7)	4 (1.4)	14 (1.9)
Sex: Female (count (%))	308 (29.5)	88 (30.4)	220 (29.2)
Sex: Male (count (%))	735 (70.5)	201 (69.6)	534 (70.8)
Race: White (count (%))	716 (68.6)	232 (80.3)	484 (64.2)
Race: Black (count (%))	11 (1.1)	1 (0.3)	10 (1.3)
Race: Asian (count (%))	62 (5.9)	9 (3.1)	53 (7)
Race: Other (count (%))	10 (1)	4 (1.4)	6 (0.8)
Time since first diagnosis (Days) (mean, 95%CI)	16.8 (15.9–17.8)	16.6 (14.9–18.4)	16.9 (15.8–18.1)
Laboratory Findings			
Haemoglobin (G/L) (mean, 95%CI)	99.5 (96.1–102.9)	110.1 (104.5–115.7)	95.4 (91.2–99.5)
Neutrophils (10^9^/L) (mean, 95%CI)	6.4 (6.1–6.6)	6.5 (6.1–6.9)	6.3 (6–6.6)
Platelets (10^9^/L) (mean, 95%CI)	318.5 (310.5–326.5)	329.8 (314.4–345.2)	314.1 (304.7–323.5)
Leukocytes (10^9^/L) (mean, 95%CI)	9.3 (9–9.5)	9.5 (9.2–9.9)	9.2 (8.9–9.5)
Creatinine (µMol/L) (mean, 95%CI)	77.5 (76.1–78.9)	76.6 (74–79.1)	77.9 (76.3–79.6)
Lactate Dehydrogenase (U/L) (mean, 95%CI)	598.1 (537.1–659.1)	630.7 (507.2–754.2)	585.1 (515.1–655.1)
Sodium (mMol/L) (mean, 95%CI)	137.9 (137.4–138.3)	137.7 (136.9–138.6)	137.9 (137.4–138.4)
Total Bilirubin (µMol/L) (mean, 95%CI)	8.8 (8.4–9.2)	8.6 (7.8–9.3)	8.9 (8.5–9.4)
Albumin (G/L) (mean, 95%CI)	37.5 (37–38)	38 (37.1–39)	37.3 (36.7–37.9)
Alkaline Phosphatase (U/L) (mean, 95%CI)	148.9 (138.9–158.8)	147.7 (133.2–162.2)	149.3 (136.7–162)
Aspartate Aminotransferase (U/L) (mean, 95%CI)	35.3 (32.9–37.8)	34.5 (30–39)	35.7 (32.7–38.7)
Alanine Aminotransferase (U/L) (mean, 95%CI)	34.9 (32.7–37.2)	34.7 (30.4–39)	35 (32.3–37.7)
Concomitant Medications			
Analgesic (count (%))	353 (33.8)	78 (27)	275 (36.5)
Blood Agents (count (%))	98 (9.4)	14 (4.8)	84 (11.1)
Anti-Inflammatory (count (%))	322 (30.9)	73 (25.3)	249 (33)
GI Tract (count (%))	332 (31.8)	76 (26.3)	256 (34)
Hypertension (count (%))	215 (20.6)	42 (14.5)	173 (22.9)
Respiratory (count (%))	240 (23)	51 (17.6)	189 (25.1)
Nitrate (count (%))	23 (2.2)	2 (0.7)	21 (2.8)
Diabetes (count (%))	50 (4.8)	12 (4.2)	38 (5)
Vaso acting (count (%))	14 (1.3)	4 (1.4)	10 (1.3)
Osteoporosis (count (%))	27 (2.6)	7 (2.4)	20 (2.7)
Brain and Mind (count (%))	234 (22.4)	50 (17.3)	184 (24.4)
Statin (count (%))	63 (6)	12 (4.2)	51 (6.8)
Gout (count (%))	32 (3.1)	11 (3.8)	21 (2.8)
Infections (count (%))	100 (9.6)	26 (9)	74 (9.8)
Cardiac (count (%))	25 (2.4)	3 (1)	22 (2.9)
Thyroid (count (%))	29 (2.8)	2 (0.7)	27 (3.6)
Cancer (count (%))	21 (2)	3 (1)	18 (2.4)
Muscle relaxant (count (%))	11 (1.1)	2 (0.7)	9 (1.2)

95 % CI, refers to the 95% confidence intervals. For continuous features the values inside the brackets are the 95% CIs. For the categorical features, the values inside the brackets are the number of patients who fall into a specific group, expressed as a percentage.

Only baseline features were included for the analysis in order to assess whether the model is able identify patients who are at risk of developing a particular SAE group and grade before they commence their treatment. Typically, age is often displayed as a continuous feature. However, for some of the clinical trials only an estimated age range was provided, thus in order to preserve as much data as possible all ages were based on ranges. For the concomitant medications, the groups were based on the main indication for each of the medications that were supplied. Many of the concomitant entries for the patients had missing entries for the intended indication of the concomitant medication. Where medications had multiple indications, they were placed in a separate group. An example of this is the nitrates group which refers to medications such as glyceryl trinitrate which can be used for cardiac therapy as well as blood pressure (Hashimoto and Kobayashi, 2003). Vaso acting medications such as pentoxifylline refers to treatments that also can be used to treat both blood pressure and cardiac therapy, however, they may have a different mechanism when compared to the nitrates class (Hashimoto and Kobayashi, 2003; Prasad and Lee, 2007). Features that had more than 80% of entries missing were excluded from the analysis. The correlation values for each of the features when compared to the onset of any SAE can be found in Supplementary Table S1 of the Supplementary Materials.

### 2.2 SAE occurrence and common SAE groups

As mentioned in the introduction, SAE refers to an adverse event that is grade 3 or higher. Out of 1,043 patients, 754 patients experienced a SAE (Table 1), the most common SAE was neutropenia, which accounted for 37% of all the SAE occurrences during the prescribed chemotherapy treatment. The three most common SAE groups were Blood and Lymphatic Disorders (59.4% of entries), Infections and Infestations (7.3% of entries) and Metabolism and Nutrition Disorders (5.6% of entries).

### 2.3 Development of machine learning models

#### 2.3.1 Preparation of data for machine learning

Different analysis groups were formed based on the three common SAE groups. All of the analysis groups contained SAE information as well as the features (variables) that are presented in Table 1. In order to handle the multiclassification of different SAE grades and groups, a 1 vs. 1 approach was used. An example of the 1 vs. 1 approach would be patients who experienced a Blood and Lymphatic Disorder SAE grade 3 vs. patients who experienced a Blood and Lymphatic Disorder SAE grade 4. The model would then predict whether patients experienced grade 3 or grade 4. From the three common SAE groups a total of 12 analysis groups were formed. Analysis groups which resulted in less than 100 patients in total were excluded as it was deemed that there would be insufficient information available for the machine learning to make robust predictions. An example of one of the analysis groups that was excluded was Blood and Lymphatic Disorder SAE grade 5 vs. Infections and Infestations SAE grade 5.

While the Blood and Lymphatic SAE group accounted for 54.7% of SAE entries, other SAE groups (as well as higher grades) are likely to have fewer individuals who experienced that particular SAE group and grade. This would result in an imbalanced training set which could decrease the model’s ability and performance. Many models that train on an imbalanced dataset will most likely predict the majority class as there are more instances present in the data set than for the minority class. There are several methods for handling imbalanced datasets, such as the inclusion of weights, costs, and sampling techniques (Blagus and Lusa, 2013; Dubey et al., 2014; Tao et al., 2019). The synthetic minority oversampling technique (SMOTE) was applied to the training set in order to balance the number of cases for both the majority and minority class. SMOTE creates new instances of the minority class, as well as reducing the number of entries in the majority class (Blagus and Lusa, 2013). The term “class” simply refers to the target variable which for all the analysis groups will be the SAE groups and grades that are compared to each other using the 1 vs. 1 approach. Other techniques were attempted such as using weights, costs, up-sampling, and down-sampling. Models that were trained with SMOTE, in this analysis always achieved higher performance scores when compared to the other techniques.

Table 2 shows the different subdivisions of the data that were used in order to develop the machine learning models.

**TABLE 2:** Summary of the different analysis groups used to build the machine learning models.

Analysis group	Original training set (80%)	SMOTE training set	Testing set (20%)	Positive class % in SMOTE training set	Positive class % in testing set
Blood 0 vs. 3	461	1,596	115	57	47
Blood 0 vs. 4	443	1,442	110	43	53
Blood 3 vs. 4	441	1,463	110	43	50
Infec 0 vs. 3	295	455	73	43	19
Infec 0 vs. 4	254	140	63	43	13
Infec 0 vs. 5	244	77	60	43	7
Infec 3 vs. 4	86	154	21	43	29
Metab 0 vs. 3	272	322	68	43	7
Metab 0 vs. 4	245	77	61	43	10
Blood vs. Infec	244	238	61	43	7
Blood vs. Metab	240	119	60	43	3
Infect vs. Metab	90	245	22	43	32

Blood: Blood and Lymphatic Disorder group, Infec: Infections and Infestations SAE, group. Metab: Metabolism and Nutrition Disorder group. The numbers in the analysis group refer to the SAE, grade. Note that for SAE, 0, this refers to individuals who developed no SAE during the trial. For the last three analysis group, e.g., blood vs. infec, the SAE, grade was 3, for instance Blood group grade 3 vs. Infec group grade 3. SMOTE: synthetic minority oversampling technique. Positive class refers to the group that is on the right side of the vs., for instance in the first group the positive group is Blood group with SAE, grade 3.

The analysis groups were then split into an 80:20 split for the training and testing datasets. This decision was made so that the model was able to learn as much as possible from the training set. After splitting the data and transforming the training data through SMOTE, the K nearest neighbours (KNN) algorithm was then applied in order to predict values for any missing data entries. In short the KNN algorithm predicted the missing value entry based on the values of its corresponding neighbours (Malarvizhi and Thanamani, 2012). The features were then centred and normalised in order to minimise the likelihood that the model will favour particular features because they seem larger in absolute value when compared other features. Once these implementations were completed, a machine learning algorithm can be applied to the training data, in order to learn any intricate patterns between the features and the target variable.

#### 2.3.2 AI implementation: extreme gradient boosting

In this analysis, the algorithm of choice used for the machine learning was the extreme gradient boosting (XGBOOST) algorithm. Other algorithms were tested such as random forest, decision trees and neural networks. However, XGBOOST yielded the best results in terms of resulting values for the area under the curve (AUC) and sensitivity rates. XGBOOST is a sequential gradient boosting algorithm developed by Tianqi Chen. This algorithm can be used for both classification and regression problems (Chen and Guestrin, 2016). In classification problems, the target variable is often presented as a 0 or a 1. A simplified example of how XGBOOST predicts the target variable is presented in the following.

In the example training data, the target variable is Blood and Lymphatic Disorders SAE grade 0 vs. grade 3. A “0” in the target variable would indicate patient did not develop any SAE whereas “1” would indicate a patient did develop a Blood and Lymphatic Disorder SAE grade 3. There are five patients in this example, three of them did develop Blood and Lymphatic Disorders SAE grade 3 and two of the patients did not. Therefore, three of the patients have “1” as their target label and the other patients have “0” as their target label.

The base XGBOOST model for binary classification will predict 0.5 for all patients. Based on this, residuals can be calculated in order to take into account the difference between the base predictions and the true target label. As an example, the residuals for the patients who did not develop SAE grade 3 would be −0.5 (0–0.5). Once the residuals were calculated then the similarity score can be obtained. This equation is given by:

$$Similarity\;Score=\frac{{\left(\sum residuals\right)}^{2}}{\sum \left[{previous\;probability}_{i}\times \left(1-previous\;{probability}_{i}\right)\right]+Regularisation\;parameter\;\left(\lambda \right)}$$ (1)

where, in this case, the previous probability refers to the base probability. The regularisation parameter λ can be used to determine whether more branches (splits) should be developed for this model (tree pruning). Assuming that λ is set to 1, the similarity score in this example is 0.111.

This similarity score is then compared to a new similarity score which has been formed based on the addition of a feature. For this example, the feature that was implemented was age. Patients who were younger than 50 years old fell into one group whereas those who were aged 50 or older were placed into a different group. The similarity scores for both of these groups were calculated. For simplicity, the similarity score for patients who are younger than 50 years old was 0.8 and the similarity score for the other group was 1. These two similarity scores were then compared to the previous score in order to calculate the gain.

$$Gain=\left(Sum\;of\;the\;Similarity\;Score\;after\;split\right)-\left(Sum\;of\;the\;Similarity\;Score\;before\;split\right)$$ (2)

The gains here are 0.689 and 0.889 respectively. As these values are positive this split is feasible. If the maximum number of splits has not been achieved then the model can continue to branch out and incorporate different features. However, in this example the maximum split has been achieved and thus the new predictions can now be formed. First the output value is calculated which is given by:

$$Output\;Value=\frac{\sum Residual\;Errors}{\sum \left[{Previous\;Probability}_{i}\times \left(1-previous\;{probability}_{i}\right)\right]+Regularisation\;parameter\;\left(\lambda \right)}$$ (3)

Note that the output values are now based on the age groups as well as the initial target variable. The equations for the new predictions and prediction probabilities are given below:

$$New\;Predictions=\left(initial\;predictions+learning\;rate\times Output\;values\right)$$ (4)

$$New\;predictions\;Probability=\frac{e^{New\;Predictions}}{1+e^{New\;Predictions}}$$ (5)

where the learning rate (which impacts on the step size of each iteration) is often given by 0.3. With the new predictions any new iterations that are made will build upon the probabilities from the previous iterations with the aim of reducing the residuals to 0.

In this analysis, the negative log likelihood of each of the iterations is used to measure the performance of each iteration. The negative log likelihood takes into account the new prediction probabilities and the actual true labels (0 and 1). Therefore, by reducing the negative log likelihood the residuals of the model are also reduced and the accuracy of the model is improved.

#### 2.3.3 Optimal parameter values for the XGBOOST algorithm

Optimal values for the XGBOOST algorithm’s parameters can be obtained through hyper parameter tuning and cross validation. Such parameters include: the maximum number of splits (tree depth) and the number of features that can be used in a single iteration (known as colsample by tree in R). In hyper parameter tuning, optimal values can be found through different search methods. In this analysis, the method for finding optimal values was based on a random search, thus random combination values were used and those that yielded the best sensitivity and specificity rates were used for the training of the model. In cross validation, the maximum number of iterations was also established. This was based on splitting the data into 5 subsets and testing within the training data, as to whether the model would be able to predict accurate responses or not. The iteration which had the lowest negative log likelihood value based upon the analysis of the test data in the cross validation, was selected as the optimal iteration number. With all the parameter values selected, the model could efficiently be built and was applied to the testing data.

#### 2.3.4 Preparation of the testing data

The testing data does not undergo any imbalance transformation. However, the testing data do undergo missing value imputation and normalisation using the same processes as its training data counterpart. The model trained on the training data set was then used on the test data to predict the target variables for the patients.

### 2.4 Feature importance using shapley additive explanation values

Although XGBOOST trees can be displayed to highlight which features influenced the model’s decision to predict a particular output, the reality is that for complex models, there may be many trees which have multiple branches with different threshold values. This can make the overall output diagram challenging to interpret. An alternative approach is to compute the Shapley additive explanatory (SHAP) values, in order to assess which features contributed the most to the model predictions (Hart, 1989; Li et al., 2020). SHAP values are based on Game Theory, where each feature value has a contribution score to the overall model’s response. This contribution score is based on the impact a specific feature value has on the model predictions and, the impact the feature value has in combination with other feature values, on the model predictions. The contribution score as well as the initial model’s bias (0.5) are summed to yield final predicted score for each patient. Using the Blood and Lymphatic Disorders group SAE grade 0 vs. grade 3 as an example, the SHAP values that are lower than 0 would denote a decreased risk of developing SAEs. SHAP values that are greater than 0 denote an increased risk of developing SAE grade 3.

All of the analysis was performed using the software tool R using the following packages for the model implementation: xgboost (XGBOOST algorithm, training the data, cross validation), caret (splitting the data, missing data implementation and normalisation), mlr (hyper-parameter tuning), RANN (necessary for knn implementation), Dmwr (SMOTE implementation), and Proc (ROC curve analysis) (R Core Team, 2023; Chen, 2023; Kuhn, 2023; Bischl, 2016; Arya et al., 2019; Torgo, 2010; Robin, 2011). The relevant codes used for this analysis can be found at: https://github.com/LindaWanika/SCLC-common-SAE-groups.

## 3 Results

### 3.1 Optimal iteration number for each of the models

**FIGURE 1:**
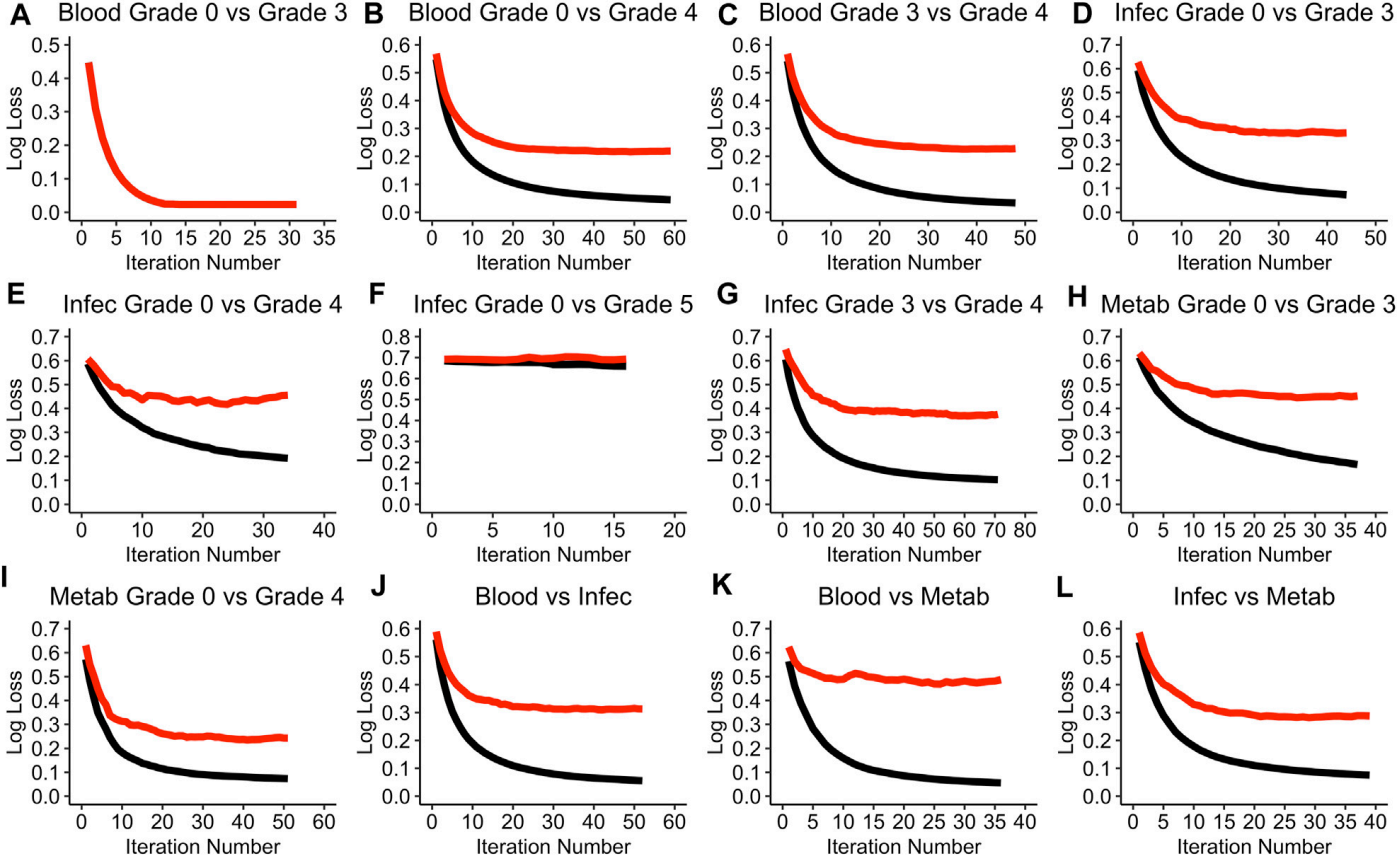
Performance of each of the models during the cross-validation process. **(A)** Blood Grade 0 vs Grade 3 model. **(B)** Blood Grade 0 vs Grade 4 model. **(C)** Blood Grade 3 vs Grade 4 model. **(D)** Infec Grade 0 vs Grade 3 model. **(E)** Infec Grade 0 vs Grade 4 model. **(F)** Infec Grade 0 vs Grade 5 model. **(G)** Infec Grade 3 vs Grade 4 model. **(H)** Metab Grade 0 vs Grade 3 model. **(I)** Metab Grade 0 vs Grade 4 model. **(J)** Blood vs Infec model. **(K)** Blood vs Metab model. **(L)** Infec vs Metab model. A black line refers to the training log loss and a red line refers to the test log loss evaluation. Note that the term “test” does not refer to the testing data set but rather the cross-validation test data. Blood: Blood and Lymphatic Disorder group, Infec: Infections and Infestations SAE group. Metab: Metabolism and Nutrition Disorder group. Log loss refers to the negative log likelihood.

Figure 1 visualises the cross-validation process for each of the models.

The Blood and Lymphatic Disorders group, SAE grade 0 vs. grade 3 model (Blood grade 0 vs. grade 3), appears to be the only model in the cross-validation process where the log loss value is able to reach to 0 for both the training and testing evaluation (see Figure 1A). The Infections and Infestation group, SAE grade 0 vs. grade 5 (Infec grade 0 vs. grade 5) has the highest negative loglikelihood (log loss) value of 0.7 even after the ideal iteration number has been given (Figure 1F). In most of the model evaluations, it is apparent that the training evaluation performs better than the testing evaluation, moreover, most of the training evaluations are able to achieve a log loss of approximately 0. A summary of all the parameter values that were chosen for each of the models based on the random search during the hyper tuning process can be found in Supplementary Table S2 in the Supplementary Materials. Table 3 summarises the optimal iteration number for each of the models.

**TABLE 3:** Summary of the best iterations for each of the models based on the cross-validation test evaluation.

Models number*	1	2	3	4	5	6	7	8	9	10	11	12
Best iteration	21	49	38	34	24	6	61	27	41	42	26	29

The model number refers to the order that they appear in Figure 1. For example, model 1 is Blood grade 0 vs. 3, model 2 is Blood grade 0 vs. grade 4, etc.

The Infec grade 0 vs. grade 5 model has the least number of iterations needed whereas the Infections and Infestations group SAE grade 3 vs. grade 4 (Infec grade 3 vs. grade 4), has the highest iteration number (Table 3).

### 3.2 Comparisons of the average test runs

**FIGURE 2:**
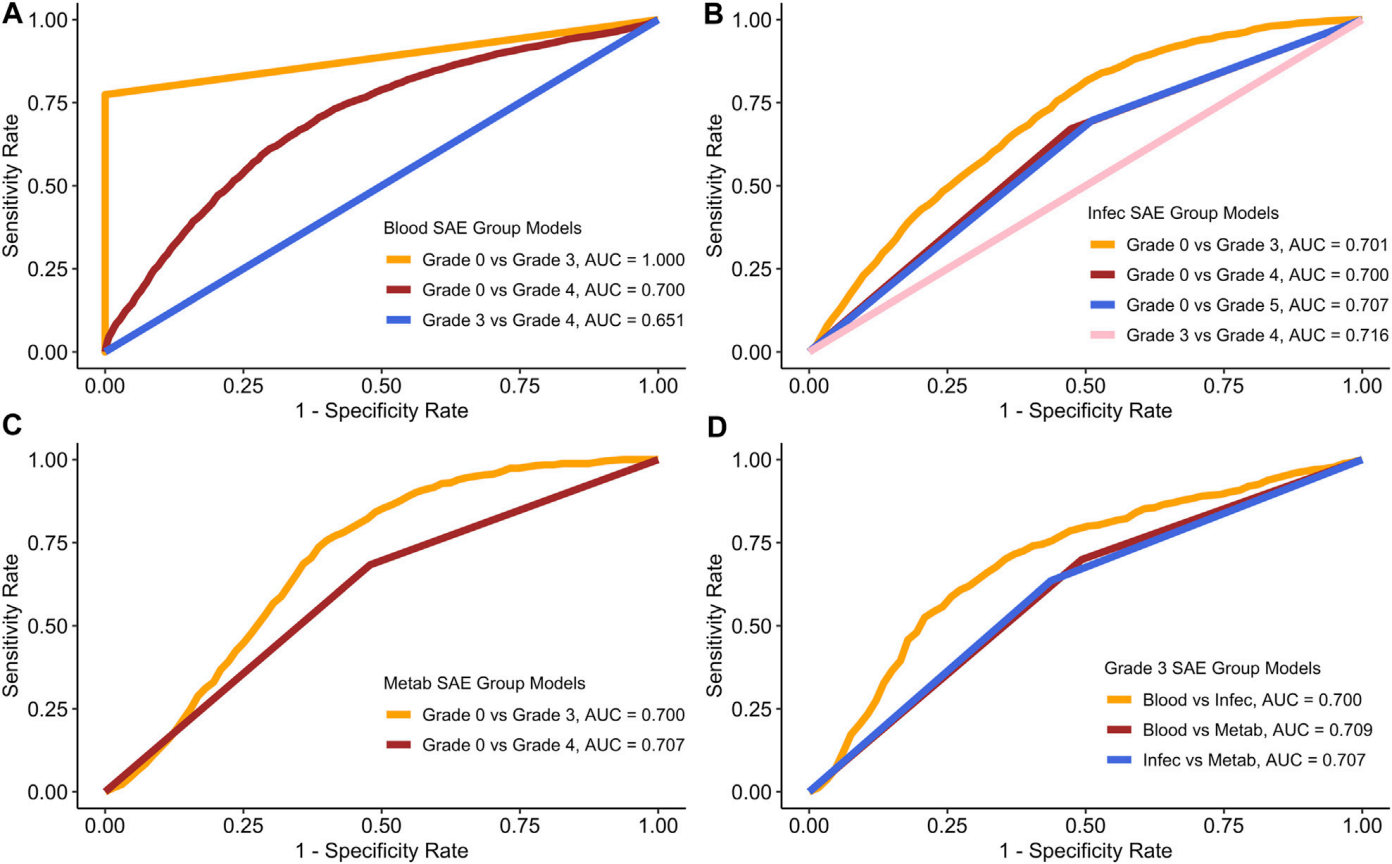
ROC curves for each of the models based on the average model run. **(A)** Blood SAE group models. **(B)** Infec SAE group models. **(C)** Metab SAE group models. **(D)** Grade 3 SAE group models. Blood: Blood and Lymphatic Disorder group, Infec: Infections and Infestations SAE group. Metab: Metabolism and Nutrition Disorder group.

Figure 2 displays the average receiver operating haracteristic (ROC) curves for each of the models.

Figure 2 shows the average AUC, sensitivity and specificity scores for the testing data based on 100 model runs. Table 4 summarises all the scores.

**TABLE 4:** Average results from all 100 testing runs for each model.

Models	AUC	Sensitivity	Specificity
Blood Group SAE 0 vs. 3	1.000	0.774	0.896
Blood Group SAE 0 vs. 4	0.700	0.594	0.406
Blood Group SAE 3 vs. 4	0.651	0.575	0.575
Infec Group SAE 0 vs. 3	0.701	0.660	0.538
Infec Group SAE 0 vs. 4	0.700	0.671	0.527
Infec Group SAE 0 vs. 5	0.707	0.696	0.489
Infec Group SAE 3 vs. 4	0.716	0.648	0.559
Metab Group SAE 0 vs. 3	0.700	0.682	0.514
Metab Group SAE 0 vs. 4	0.707	0.683	0.521
Blood vs. Infec Group (SAE 3)	0.700	0.684	0.513
Blood vs. Metab Group (SAE 3)	0.709	0.699	0.507
Infec vs. Metab Group (SAE 3)	0.707	0.635	0.563

AUC: area under the curve. Blood: Blood and Lymphatic Disorder group, infec: Infections and Infestations SAE, group. Metab: Metabolism and Nutrition Disorder group.

In both Figure 2A and in Table 4, the Blood grade 0 vs. grade 3 model, has the highest AUC and on average the highest sensitivity and specificity rates. The Blood group grade 3 vs. grade 4 has, on average have the lowest AUC, and sensitivity rate (Figure 2A). However, the Blood grade 0 vs. grade 4, on average has the lowest specificity rate at 0.406 (Table 3). The other models in comparison, appear to have similar AUC scores on average, at approximately 0.7 (Figures 2B–D).

### 3.3 Comparisons of the best test runs

**FIGURE 3:**
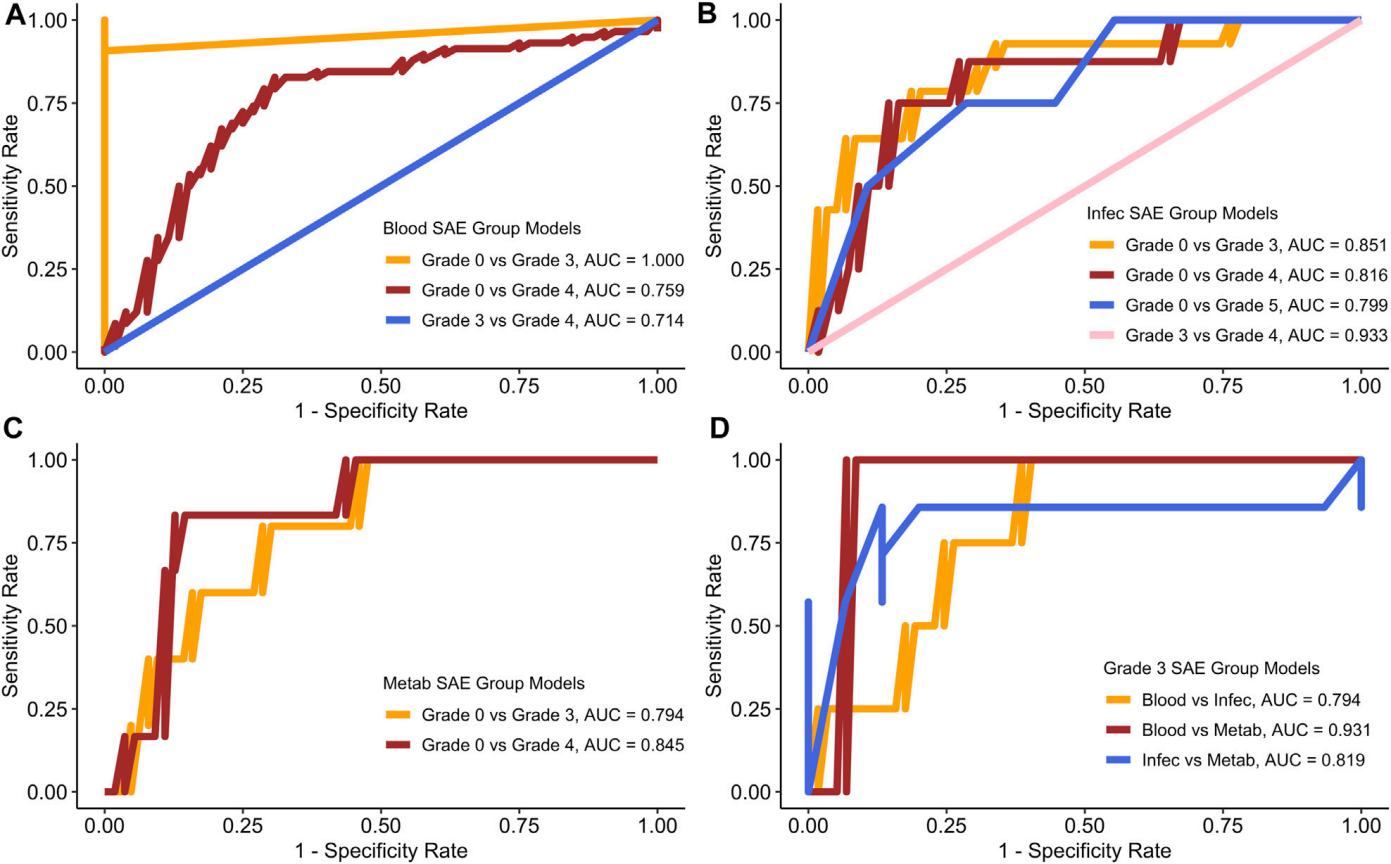
ROC curves for each of the models based on the best test run. **(A)** Blood SAE group models. **(B)** Infec SAE group models. **(C)** Metab SAE group models. **(D)** Grade 3 SAE group models. Blood: Blood and Lymphatic Disorder group, Infec: Infections and Infestations SAE group. Metab: Metabolism and Nutrition Disorder group.

Figure 3 displays the best ROC curves for each of the models.

Figure 3 shows that the sensitivity and specificity rates often fluctuate. Table 5 summarises all the scores for the best test runs for each of the models.

**TABLE 5:** Best test runs for each model.

Models	AUC	Sensitivity	Specificity
Blood Group SAE 0 vs. 3	1.000	0.840	0.964
Blood Group SAE 0 vs. 4	0.759	0.621	0.635
Blood Group SAE 3 vs. 4	0.714	0.606	0.606
Infec Group SAE 0 vs. 3	0.851	0.780	0.566
Infec Group SAE 0 vs. 4	0.816	0.771	0.582
Infec Group SAE 0 vs. 5	0.799	0.828	0.621
Infec Group SAE 3 vs. 4	0.933	0.795	0.618
Metab Group SAE 0 vs. 3	0.794	0.768	0.521
Metab Group SAE 0 vs. 4	0.845	0.806	0.533
Blood vs. Infec Group (SAE 3)	0.794	0.770	0.519
Blood vs. Metab Group (SAE 3)	0.931	0.910	0.514
Infec vs. Metab Group (SAE 3)	0.819	0.708	0.597

AUC: area under the curve. Blood: Blood and Lymphatic Disorder group, infec: Infections and Infestations SAE, group. Metab: Metabolism and Nutrition Disorder group.

Similar to the values generated for the average run, the Blood group grade 0 vs. grade 3 also had the best AUC sensitivity and specificity rates (Figure 3A; Table 5). In the Infections and Infestations group analysis (Figure 3B), the average AUC score for all the models is 0.8 with the grade 3 vs. grade 4 group having the highest AUC at 0.933. For the Metabolism and Nutrition Disorders group analysis (Figure 3C), the average AUC is also 0.8 and both of the models yield a higher sensitivity rate than specificity (Table 5). In the combinations group analysis (Figure 3D), the Blood vs. Metabolism analysis yields the highest AUC and sensitivity rates out of all the models in general with a score of 0.910.

The confusion matrices for both the average test runs and the best testing runs can be found in Supplementary Tables S3, S4 in the Supplementary Materials.

### 3.4 Feature analysis for all of the models

**FIGURE 4:**
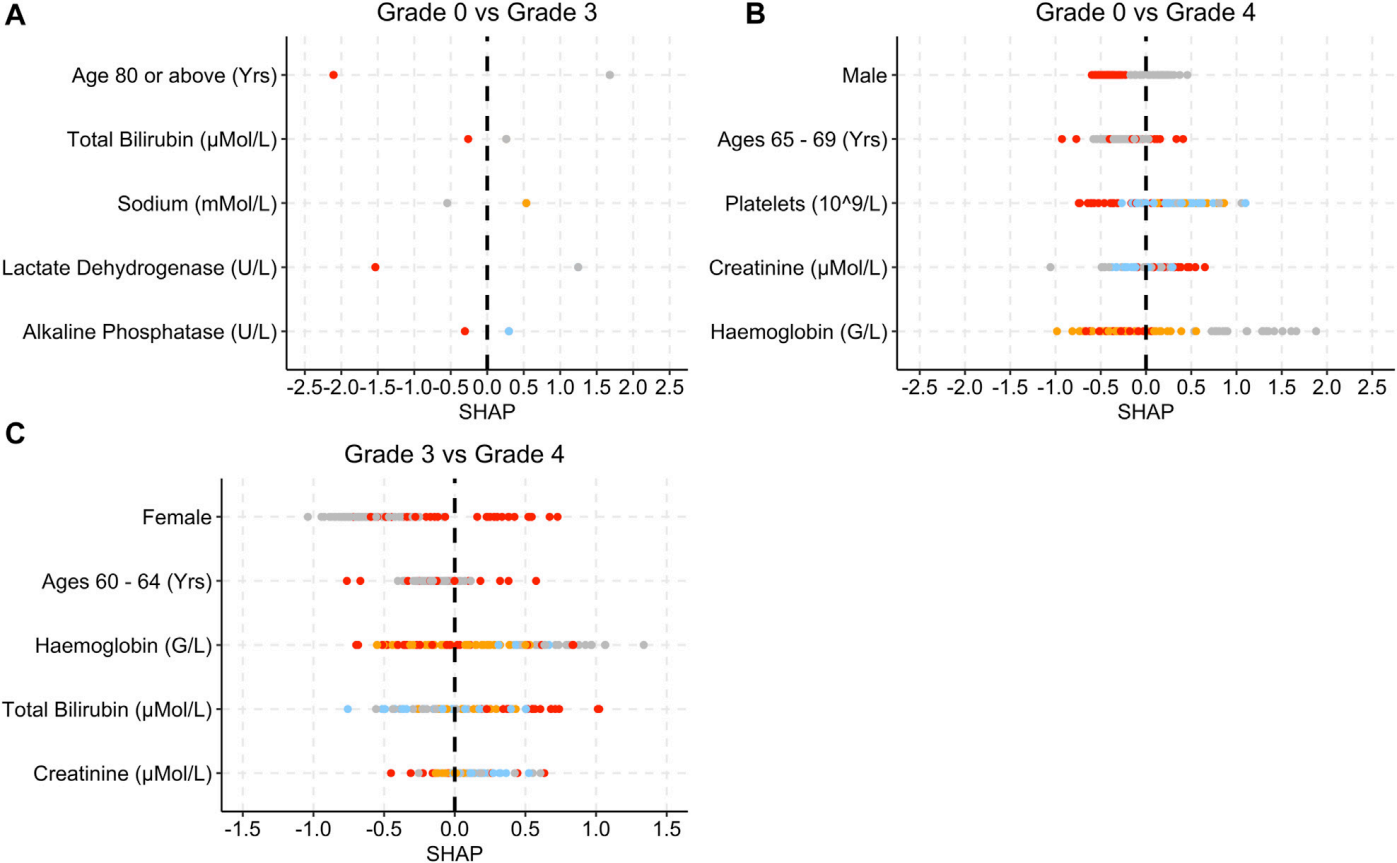
SHAP plots displaying the top five features for the Blood group SAE models. **(A)** Grade 0 vs Grade 3 model. **(B)** Grade 0 vs Grade 4 model. **(C)** Grade 3 vs Grade 4 model. Grey dots refer to feature values that are below the lower quartile range (LQR), blue dots refer to the feature values that fall between LQR and the mean. Orange dots refer to the feature values that fall between the mean and upper quartile range (UQR). Red dots refer to values that are above the UQR.

Figure 4 provides the SHAP plots for the Blood and Lymphatic Disorders group SAE models.

For the grade 0 vs. grade 3 model, patients who were over 80 years old, had high total bilirubin, lactate dehydrogenase and alkaline phosphatase levels, obtained SHAP values that were below 0 (Figure 4A). In Figure 4B, female patients, low platelet, and haemoglobin levels as well as high creatinine levels yielded SHAP values that were above 0 for grade 0 vs. grade 4. In Figure 4C, patients who were female and had a higher total bilirubin level obtained higher SHAP values, whereas patients who had higher creatinine levels obtained SHAP values less than 0.

**FIGURE 5:**
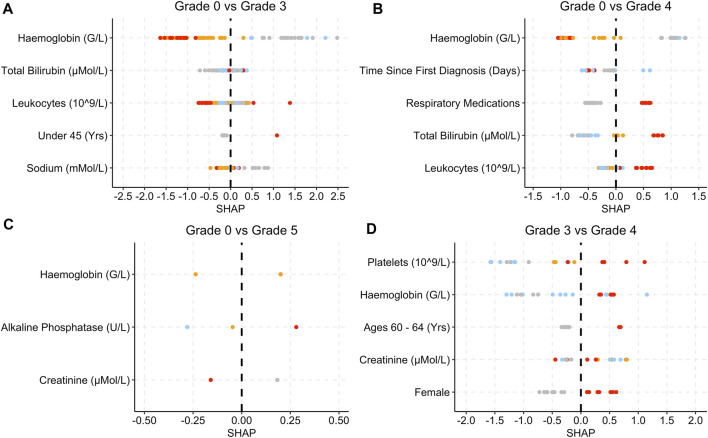
SHAP plots displaying the top five features for the Infec group SAE models. **(A)** Grade 0 vs Grade 3 model. **(B)** Grade 0 vs Grade 4 model. **(C)**: Grade 0 vs Grade 5 model. **(D)** Grade 3 vs Grade 4 model. Grey dots refer to feature values that are below the lower quartile range (LQR), blue dots refer to the feature values that fall between LQR and the mean. Orange dots refer to the feature values that fall between mean the and upper quartile range (UQR). Red dots refer to values that are above the UQR. For Grade 0 vs Grade 5, only three features were used to perform the predictions.

Figure 5 provides the SHAP plots for the Infections and Infestations group SAE models.

For the grade 0 vs. grade 3 model, low haemoglobin levels, patients who were under 45 years old and low sodium levels were associated with SHAP values that are above 0 (Figure 5A). Low haemoglobin levels are also associated with SHAP values that are less than 0 for the grade 0 vs. grade 4, grade 0 vs. grade 5 and grade 3 vs. 4 models (Figure 5). High alkaline phosphate levels are associated with grade 5 and only three features were used in total for the prediction (Figure 5C). Respiratory medications and high total bilirubin and leukocytes levels are associated with higher SHAP values (Figure 5B).

**FIGURE 6:**
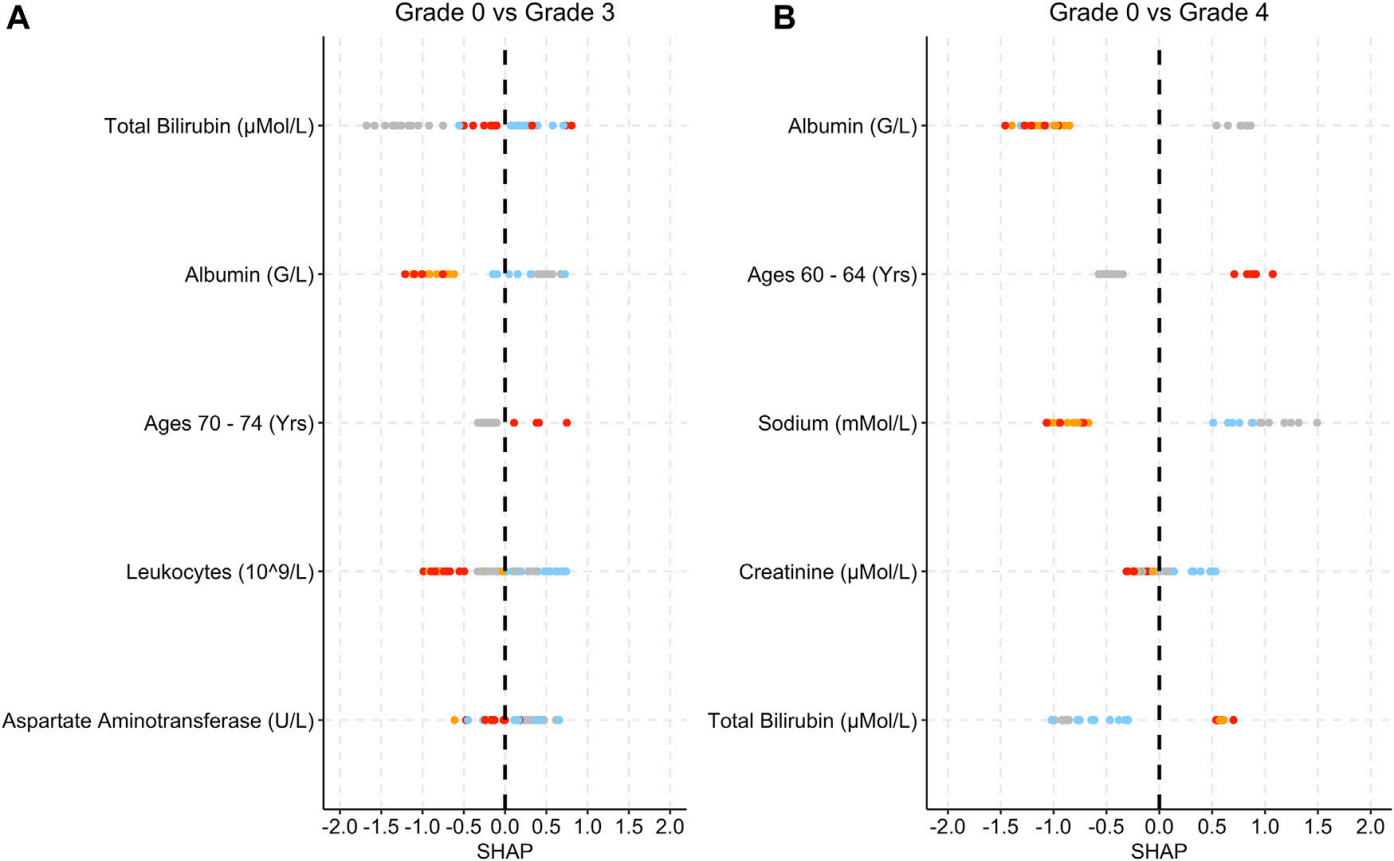
SHAP plots displaying the top five features for the Metab group SAE models. **(A)** Grade 0 vs Grade 3 model. **(B)** Grade 0 vs Grade 4 model. Grey dots refer to feature values that are below the lower quartile range (LQR), blue dots refer to the feature values that fall between LQR and the mean. Orange dots refer to the feature values that fall between the mean and upper quartile range (UQR). Red dots refer to values that are above the UQR.

Figure 6 provides the SHAP plots for the Metabolism and Nutrition Disorders group SAE models.

Low albumin levels are associated with higher SHAP values (Figure 6). High leukocyte levels in grade 0 vs. grade 3 are associated with low SHAP values (Figure 6A). For both models, lower bilirubin levels are associated with SHAP values below 0.

**FIGURE 7:**
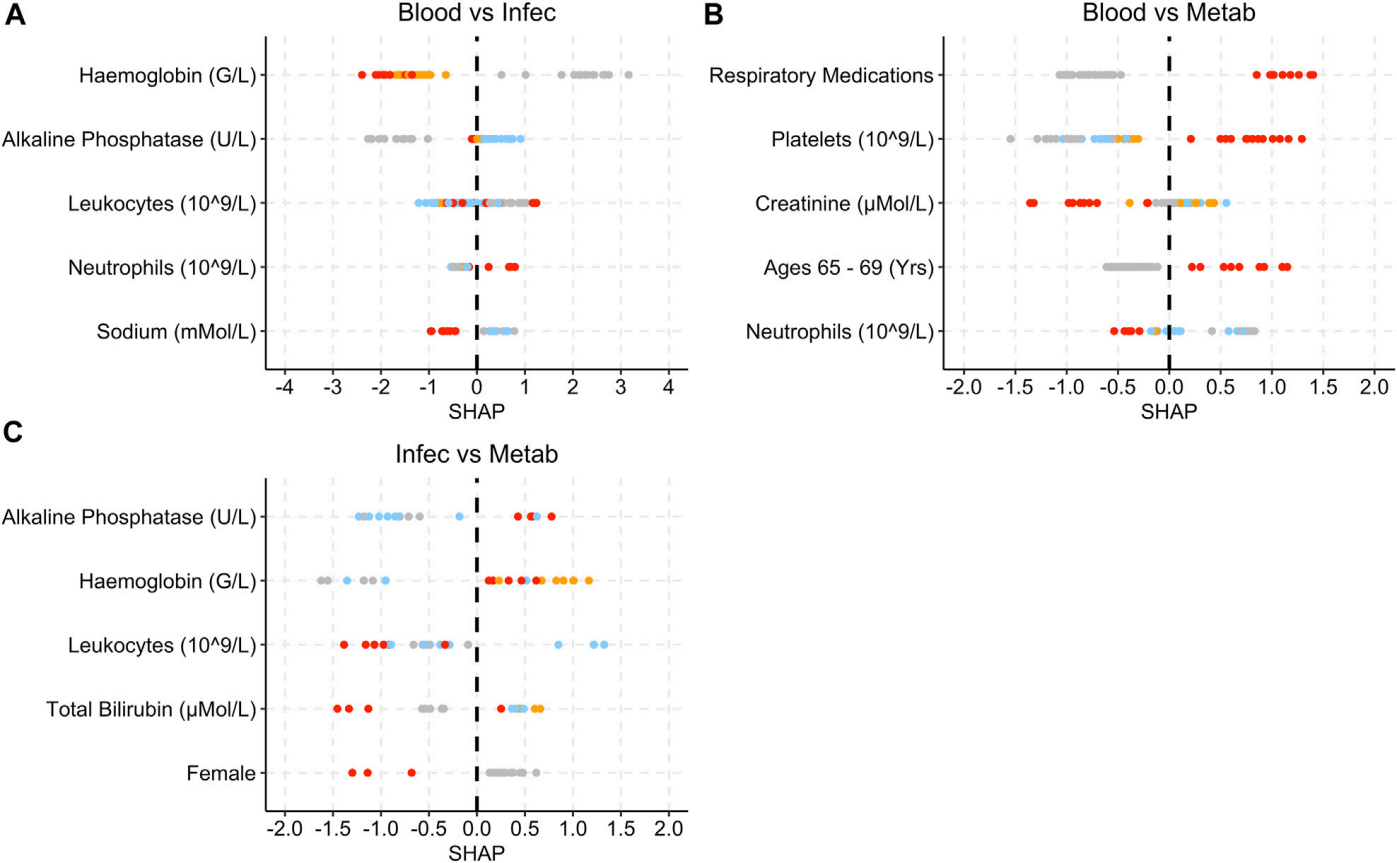
SHAP plots displaying the top five features for the grade 3 SAE group models. **(A)** Blood vs Infec model. **(B)** Blood vs Metab model. **(C)** Infec vs Metab model. Grey dots refer to feature values that are below the lower quartile range (LQR), blue dots refer to the feature values that fall between LQR and the mean. Orange dots refer to the feature values that fall between the mean and upper quartile range (UQR). Red dots refer to values that are above the UQR.

Figure 7 provides the SHAP plots for the comparison SAE groups.

For the Blood and Lymphatic Disorders group vs. Infections and Infestations group, high haemoglobin and sodium levels are associated with SHAP values below 0, whereas high neutrophils are associated with higher SHAP values (Figure 7A). For the Blood and Lymphatic Disorders group vs. Metabolism and Nutrition Disorders group, patients who had respiratory medications, high platelet levels and patients who were aged between 65 and 69 years were associated with high SHAP values, low neutrophils were associated lower SHAP values. In Figure 7C, high alkaline phosphatase and haemoglobin were associated with high SHAP values whereas high leukocytes and total bilirubin and patients who are female were associated with low SHAP values.

## 4 Discussion

Based on the average model analysis, most of the models were able to correctly identify a higher number of patients who fell into the more severe group of the 1 vs. 1 analysis group compared to the number of patients who fell into the less severe group. This is evidenced in Table 4, with most of the average models having a higher sensitivity rate than specificity rate. Sensitivity rates measures the “true positive rates”, i.e., classes which fall to the right side of the vs. group, whereas specificity rates measure the “true negative rate”, which are classes which fall on the left side of the vs. group. In a real-world setting, this outcome is more beneficial as misclassifying a patient as at risk of developing SAE grade 4 when in reality they are not at risk of developing any SAE, is a better outcome than misclassifying a patient as not at risk of developing any SAE, when in fact the patient is at risk of developing SAE grade 4. However, for the comparison models between different groups, both sides in the analysis group have the same level of severity. A possible reason as to why more patients were predicted as at risk in the “positive” group could be that the model was trained on a training set where 43% of cases were positive, however the actual test set had less than 50% of “positive” cases for the Infections and Infestations group, the Metabolism and Nutrition Disorder group and the comparison models between different groups (Table 2). It is possible that the model predicted more positive cases simply because it assumes that more positive cases should exist.

In Table 5, the best test runs for each of the models were able to correctly classify at least 60% of patients who fell into the positive group and at least 50% of patients who fell into the negative group. By far the best model was the Blood and Lymphatic Disorder group SAE grade 0 vs. grade 3 which achieved, on average, a sensitivity rate of 0.774 and a specificity rate of 0.896 (Table 4). The best run for this model, in particular, achieved a sensitivity score of 0.840 and a specificity rate of 0.964 (Table 5). It is important to note that the sensitivity and specificity scores presented are the mean and not the maximum rates. Other models that achieved high predictive scores (based on the best test runs) were the Infections and Infestations group SAE grade 0 vs. grade 5 and the Blood and Lymphatic Disorder group vs. the Metabolism and Nutrition Disorder group (Figures 2, 3; Table 5). While the Infections and Infestations group SAE grade 3 vs. grade 4 model achieved a higher AUC score (Tables 4, 5), the grade 0 vs. 5 achieved higher sensitivity and specificity rates. Moreover, this model, in particular, had fewer iterations and thus was also simpler than the grade 3 vs. grade 4 (Table 3). For the Infections and Infestation group SAE grade 0 vs. grade 5, the model was able to correctly identify 82% of patients who were at risk of developing SAE grade 5 and identified 62% of patients who are not at risk of developing SAEs (Table 5). The Blood and Lymphatic Disorder group vs. the Metabolism and Nutrition Disorder group model was able to identify 91% of patients who were at risk of developing a Metabolism and Nutrition Disorder group SAE grade 3 and identified 51% of patients who were at risk of developing a Blood and Lymphatic Disorder group SAE grade 3.

The SHAP plots for each of the models provides a simple and clear overview of the top features that contributed the most for each model prediction. The SHAP plots that are presented here are based on the best test runs for each model. Values that are above 0 indicate that patients are more at risk of being in the positive class, whereas values that are less than 0 indicate that patients are either not at risk of developing SAE (for models that compare grade 0 to another grade), or the negative class. Note that the classification of the feature values (lower quartile, mean, upper quartile) does not necessarily denote that the values are abnormal readings (see Supplementary Table S17–S28 for the summary statistics of each of the features for the respective models in the Supplementary Materials).

For the Blood and Lymphatic Disorders group analysis, patients with low haemoglobin levels were associated with being at risk of developing Blood group SAE grade 4, whereas low total bilirubin levels were associated with patients not being at risk of developing SAE (Figure 4). Low haemoglobin can also be associated with anaemia and other Blood Lymphatic Disorders (Mercadante et al., 2000; Rusciano et al., 2008). High bilirubin levels can also be associated with the breakdown of haemoglobin which may result in decreased levels of haemoglobin (Figure 4C) (Kao et al., 2012). For the first model (grade 0 vs. grade 3), patients who were aged 80 or above were deemed as less likely to develop Blood SAE. In Table 1, the majority of patients are not in this age group range and have a higher incidence of SAEs, in general. This could potentially explain as to why the model highlighted this as an important feature. For the second model (grade 0 vs. grade 4) low platelet levels were also associated with a higher risk of grade 4 which is more in keeping with what is known, as thrombocytopenia is a common SAE and is often associated with chemotherapy treatment (Weycker et al., 2019).

Similar to the Blood and Lymphatic Disorders group, in the Infections and Infestations group (Figure 5) low haemoglobin was associated with grade 3 severity, and in some instances grade 4 (Figure 5B), and low bilirubin is associated with patients less at risk of developing an SAE. Lymphatic disorders can make patients more suspectable to infections as the levels of lymphocytes decrease (Francis et al., 2013). For grade 0 vs. grade 4, patients who were taking respiratory medications and had high leukocyte levels were also more at risk of developing grade 4. A possible reason for the respiratory link to infections could be that, prior to the treatment, these patients may have been prescribed cough supplements or other respiratory medications for the treatment of respiratory conditions caused by infections (Rosen, 2006). High leukocytes also tend to be present during inflammation which may have been caused by an infection (Chmielewski and Strzelec, 2018). Higher alkaline phosphatase levels are associated with patients who are at risk of developing grade 5 (Figure 5C). High alkaline phosphatase levels can be associated with liver disorders which can also include infections (Blayney et al., 2008). For the Blood and Lymphatic Disorders group, patients who were female seem to have a higher susceptibility based on the SHAP values, to developing SAEs, even though the majority of patients who developed SAEs, in general were male (Table1). Some studies have found that females are more suspectable to infections and anaemia, as well as other blood conditions, which may be a possible reason for this difference (Nazir et al., 2011). Although Figure 5D shows females as also being a contributing factor for the Infections and Infestations group, it should be noted that this feature does not appear in any of the other Figure 5 plots.

The Metabolism and Nutrition Disorder SAE group has a smaller test set compared to the previous analysis groups (Table 2). Low albumin levels were associated with patients being at risk of grades 3 and 4 (Figure 6). Low albumin levels have been linked with hepatic disorders, which can potentially impact the metabolism process (Matthewson et al., 1986; Carvalho and Machado, 2018). This may also explain the relationship between bilirubin and the occurrence of Metabolism and Nutrition Disorder SAEs (Matthewson et al., 1986; Hamoud et al., 2018). Decreased or increased levels of minerals in the body are often associated with Metabolism and Nutrition Disorders (Cancer Therapy Evaluation Program, 2023).

In Figure 7, low haemoglobins are more associated with the Infections and Infestations group SAE grade 3 when compared to the other SAE groups. Low platelet and neutrophils levels are more indicative of Blood and Lymphatic Disorders SAE grade 3 when compared to the other groups. High alkaline phosphatase seem are associated with patients at risk of developing Metabolism and Nutrition Disorders group SAE grade 3 when compared to the Infections and Infestations group SAE grade 3 groups, as well as higher platelet levels when compared to the Blood and Lymphatic Disorders group.

The application of machine learning to this dataset has enabled the identification of trends between common SAE groups and features which may have been overlooked through the application of statistical methods alone. During the training process, XGBOOST is able to analyse multiple features and split these features accordingly in order to determine adequate feature thresholds which would impact on the predictability of common SAE group’s onset, within a short time frame (minutes). A significant amount of time would be required in order to achieve the same outcome using traditional statistical methods. Moreover, many of the traditional statistical methods rely on significant correlations between features and the predictive target. In Supplementary Table S1 (Supplementary Materials), only six features have a *p*-value of less than 0.05 when associated with the onset of SAE. Total bilirubin and alkaline phosphatase are two features which were identified as common risk factors for the onset of common SAE groups however, both of them have correlation values of less than 0.1 and *p*-values greater than 0.5.

While machine learning does have advantages in supporting model predictions, it is important to note that in order to achieve optimal results, good quality data are needed, i.e., large in quantity and a balanced dataset with minimal missing values. For adverse event onset the data are usually imbalanced given that these occurrences are generally minimal and sometimes rare. In clinical trials, and to a greater extent with real world data, missing entries are common. As mentioned in the methods section, features that had up to 80% missing entries were included in the analysis. The features that were excluded may have been significant for the onset of common SAE groups, however, it is most likely that XGBOOST would have dismissed these features. In addition to this, while KNN was used to impute the missing values, the values selected may not have been adequate. In other words, it is possible that a clinician may see the value of one feature and be able to deduce that, for another feature, values should fall within a specific range. A possible solution, when applying this technique to real world data, would be to initially assess the quality of the data and consult with clinicians to determine which features should be included and if it is possible to infer missing values from other features. From such collaboration, the techniques explained in this paper could equally be applied to study the onset of other adverse events, including rare adverse events and also the onset of other diseases.

Many of the features that are presented in the SHAP plots seem to display varied results suggesting that there is not enough evidence to suggest whether extremities of the features could be used to identify whether patients are more at risk or less at risk of developing an SAE which falls into one of these groups. An important limitation of this analysis is that the SHAP plots are only based on the best models which are based on the data provided, the data split used, and the algorithm applied. Despite using the same data split and the same parameter values there was variability within the 100 testing runs (see Supplementary Tables S16–27 in the Supplementary Materials for all 100 runs for each model). It is possible that with more runs the AUC may change and that other sets of test runs may have yielded different top five features to be explored in the SHAP plots. SHAP values are also based on an unrealistic assumption that the features are independent from each other. This assumption can lead to features being identified as providing a significantly high contribution score to the prediction when in reality it could be that certain features are always dependent on other features and this is contributing to the final contribution score (Aas et al., 2021). It is important to take into account that the results presented here are based on many factors and that the training data which the models are based on also include synthetic data (for the missing data imputation). It is therefore crucial to investigate any possible correlations between the features and predictions and perform further evaluation using statistical methods under correct assumptions in order to determine whether these features indeed have possible causative relationships with the onset of common SAE groups.

To conclude, from this study the best models for each analysis group were able to achieve sensitivity rates of at least 0.6 and AUC scores of at least 0.7. The Blood and Lymphatic Disorder group SAE grade 0 vs. grade 3 model achieved the highest AUC of 1. Other high performing models include the Infections and Infestations group SAE grade 0 vs. grade 5 and the Blood and Lymphatic Disorders group SAE grade 3 vs. the Metabolism and Nutrition Disorders group SAE grade 3. For the Blood and Lymphatic Disorder group SAE grade 0 vs. grade 3 model, patients younger than 80 years old are associated with the occurrence of grade 3. Further work should be undertaken to further investigate whether these features can be robustly used to predict the onset of these SAEs as well as identifying risk factors for other SAE groups.

## Data Availability

Pre-existing clinical data underpinning this publication are available from Project Data Sphere at https://data.projectdatasphere.org/projectdatasphere/html/access.
