# Supplementary material for: Identification of small cell lung cancer patients who are at risk of developing common serious adverse event groups with machine learning
Source: Front Drug Saf Regul. 2023 Sep 15;3:1267623. doi: 10.3389/fdsfr.2023.1267623 (PMC12443098; doi:10.3389/fdsfr.2023.1267623)
Supplement: Supplementary file 1 [file Table1.DOCX]

Supplementary Material

Identification of Small Cell Lung Cancer Patients who are at Risk of Developing Common Serious Adverse Event Groups with Machine Learning

Linda Wanika, Neil D. Evans and Michael J. Chappell

*** Correspondence:** Michael J. Chappell: m.j.chappell@warwick.ac.uk

# Correlation values between features and SAE development

Table S1: Features and correlation values to SAE occurrence.

| Features | r value | p.value |
| --- | --- | --- |
| Demographic |  |  |
| Age: below 45 Yrs (count (%)) | 0.02 | 0.6 |
| Age: 45 to 49 Yrs (count (%)) | -0.1 | 0.0005 |
| Age: 50 to 54 Yrs (count (%)) | -0.05 | 0.1 |
| Age: 55 to 59 Yrs (count (%)) | -0.02 | 0.4 |
| Age: 60 to 64 Yrs (count (%)) | 0.03 | 0.4 |
| Age: 65 to 69 Yrs (count (%)) | 0.02 | 0.6 |
| Age: 70 to 74 Yrs (count (%)) | 0.05 | 0.09 |
| Age: 75 to 79 Yrs (count (%)) | 0.06 | 0.05 |
| Age: 80 or above Yrs (count (%)) | 0.02 | 0.6 |
| Sex: Female (count (%)) | -0.01 | 0.7 |
| Sex: Male (count (%)) | 0.01 | 0.7 |
| Race: White (count (%)) | -0.1 | 0.002 |
| Race: Black (count (%)) | 0.06 | 0.1 |
| Race: Asian (count (%)) | 0.1 | 0.004 |
| Race: Other (count (%)) | -0.02 | 0.5 |
| Time since first diagnosis (Days) (mean, 95%CI) | 0.01 | 0.8 |
| Laboratory Findings |  |  |
| Haemoglobin (G/L) (mean, 95%CI) | -0.1 | 0.001 |
| Neutrophils (10^9^/L) (mean, 95%CI) | -0.04 | 0.4 |
| Platelets (10^9^/L) (mean, 95%CI) | -0.06 | 0.09 |
| Leukocytes (10^9^/L) (mean, 95%CI) | -0.04 | 0.2 |
| Creatinine (µMol/L) (mean, 95%CI) | 0.03 | 0.4 |
| Lactate Dehydrogenase (U/L) (mean, 95%CI) | -0.03 | 0.5 |
| Sodium (mMol/L) (mean, 95%CI) | 0.02 | 0.7 |
| Total Bilirubin (µMol/L) (mean, 95%CI) | 0.03 | 0.4 |
| Albumin (G/L) (mean, 95%CI) | -0.06 | 0.2 |
| Alkaline Phosphatase (U/L) (mean, 95%CI) | 0.01 | 0.9 |
| Aspartate Aminotransferase (U/L) (mean, 95%CI) | 0.02 | 0.7 |
| Alanine Aminotransferase (U/L) (mean, 95%CI) | 0 | 0.9 |
| Concomitant Medications |  |  |
| Analgesic (count (%)) | 0.04 | 0.2 |
| Blood Agents (count (%)) | 0.09 | 0.02 |
| Anti-Inflammatory (count (%)) | 0.03 | 0.5 |
| GI Tract (count (%)) | 0.02 | 0.5 |
| Hypertension (count (%)) | 0.07 | 0.07 |
| Respiratory (count (%)) | 0.04 | 0.2 |
| Nitrate (count (%)) | 0.06 | 0.08 |
| Diabetes (count (%)) | 0 | 1 |
| Vaso acting (count (%)) | -0.01 | 0.7 |
| Osteoporosis (count (%)) | -0.01 | 0.8 |
| Brain and Mind (count (%)) | 0.04 | 0.3 |
| Statin (count (%)) | 0.03 | 0.3 |
| Gout (count (%)) | -0.05 | 0.2 |
| Infections (count (%)) | -0.02 | 0.6 |
| Cardiac (count (%)) | 0.05 | 0.2 |
| Thyroid (count (%)) | 0.03 | 0.3 |
| Cancer (count (%)) | 0.08 | 0.03 |
| Muscle relaxant (count (%)) | 0.02 | 0.6 |

Values are displayed to one significant figure. r: correlation value.

# Parameter values for each of the 12 models

Table S2: Parameter values obtained during the hyper training of XGBOOST using the random search method.

| **Model Number** | **1** | **2** | **3** | **4** | **5** | **6** | **7** | **8** | **9** | **10** | **11** | **12** |
| --- | --- | --- | --- | --- | --- | --- | --- | --- | --- | --- | --- | --- |
| **Max Depth** | 3.00 | 9.00 | 8.00 | 7.00 | 4.00 | 3.00 | 3.00 | 3.00 | 7.00 | 9.00 | 8.00 | 8.00 |
| **Minimum child weight** | 7.40 | 3.29 | 1.44 | 1.63 | 9.46 | 5.28 | 2.55 | 2.64 | 1.51 | 1.83 | 1.08 | 2.44 |
| **Subsample** | 0.53 | 0.92 | 0.95 | 0.62 | 0.56 | 0.61 | 0.95 | 0.72 | 0.75 | 0.94 | 0.88 | 0.95 |
| **Colsample by tree** | 0.81 | 0.66 | 0.82 | 0.56 | 0.86 | 1.00 | 0.57 | 0.54 | 0.81 | 0.63 | 0.77 | 0.98 |

Model numbers refer to each of the models as follows; 1: Blood grade 0 vs grade 3, 2: Blood grade 0 vs grade 4, 3: Blood grade 3 vs grade 4, 4: Infec grade 0 vs grade 3, 5: Infec grade 0 vs grade 4, 6: Infec grade 0 vs grade 5, 7: Infec grade 3 vs grade 4, 8: Metab grade 0 vs grade 3, 9: Metab grade 0 vs grade 4, 10: Blood grade 3 vs Infec grade 3, 11: Blood grade 3 vs Metab grade 3 and 12: Infec grade 3 vs Metab grade 3. Max Depth refers to the maximum number of splits an iteration is permitted to have. Minimum child weight: minimum weight (denominator of the similarity score) needed in a single leaf. Subsample: % of training data that is used during the specific iteration. Colsample by tree refers to the % of features that can be used for each iteration. Values are displayed to two decimal places.

# Confusion matrices for the average test run for each of the 12 models

Table S3: Average confusion matrices for each of the 12 models.

| Models | Confusion matrices | |  |
| --- | --- | --- | --- |
| Blood 0 vs 3 | n=115 | Actual 0 | Actual 3 |
|  | Predicted 0 | 55 | 12 |
|  | Predicted 3 | 6 | 42 |
| Blood 0 vs 4 | n=110 | Actual 0 | Actual 4 |
|  | Predicted 0 | 21 | 23 |
|  | Predicted 4 | 31 | 35 |
| Blood 3 vs 4 | n=110 | Actual 3 | Actual 4 |
|  | Predicted 3 | 32 | 23 |
|  | Predicted 4 | 23 | 32 |
| Infec 0 vs 3 | n=73 | Actual 0 | Actual 3 |
|  | Predicted 0 | 32 | 5 |
|  | Predicted 3 | 27 | 9 |
| Infec 0 vs 4 | n=63 | Actual 0 | Actual 4 |
|  | Predicted 0 | 29 | 3 |
|  | Predicted 4 | 26 | 5 |
| Infec 0 vs 5 | n=60 | Actual 0 | Actual 5 |
|  | Predicted 0 | 27 | 1 |
|  | Predicted 5 | 29 | 3 |
| Infec 3 vs 4 | n=21 | Actual 3 | Actual 4 |
|  | Predicted 3 | 8 | 2 |
|  | Predicted 4 | 7 | 4 |
| Meta 0 vs 3 | n=68 | Actual 0 | Actual 3 |
|  | Predicted 0 | 33 | 2 |
|  | Predicted 3 | 30 | 3 |
| Meta 0 vs 4 | n=61 | Actual 0 | Actual 4 |
|  | Predicted 0 | 29 | 2 |
|  | Predicted 4 | 26 | 4 |
| Blood vs Infec | n=61 | Actual Blood | Actual Infec |
|  | Predicted Blood | 29 | 1 |
|  | Predicted Infec | 28 | 3 |
| Blood vs Metab | n=60 | Actual Blood | Actual Metab |
|  | Predicted Blood | 30 | 1 |
|  | Predicted Metab | 28 | 1 |
| Infec vs Metab | n=22 | Actual Infec | Actual Metab |
|  | Predicted Infec | 8 | 3 |
|  | Predicted Metab | 7 | 4 |

Predicted are the predictions from the model whereas actual refers to the true test labels.

# Confusion matrices for the best test run for each of the 12 models

Table S4: Best confusion matrices for each of the 12 models.

| Models | Confusion matrices | |  |
| --- | --- | --- | --- |
| Blood 0 vs 3 | n=115 | Actual 0 | Actual 3 |
|  | Predicted 0 | 59 | 9 |
|  | Predicted 3 | 2 | 45 |
| Blood 0 vs 4 | n=110 | Actual 0 | Actual 4 |
|  | Predicted 0 | 33 | 22 |
|  | Predicted 4 | 19 | 36 |
| Blood 3 vs 4 | n=110 | Actual 3 | Actual 4 |
|  | Predicted 3 | 33 | 22 |
|  | Predicted 4 | 22 | 33 |
| Infec 0 vs 3 | n=73 | Actual 0 | Actual 3 |
|  | Predicted 0 | 33 | 3 |
|  | Predicted 3 | 26 | 11 |
| Infec 0 vs 4 | n=63 | Actual 0 | Actual 4 |
|  | Predicted 0 | 32 | 2 |
|  | Predicted 4 | 23 | 6 |
| Infec 0 vs 5 | n=60 | Actual 0 | Actual 5 |
|  | Predicted 0 | 35 | 1 |
|  | Predicted 5 | 21 | 3 |
| Infec 3 vs 4 | n=21 | Actual 3 | Actual 4 |
|  | Predicted 3 | 9 | 1 |
|  | Predicted 4 | 6 | 5 |
| Meta 0 vs 3 | n=68 | Actual 0 | Actual 3 |
|  | Predicted 0 | 34 | 1 |
|  | Predicted 3 | 39 | 4 |
| Meta 0 vs 4 | n=61 | Actual 0 | Actual 4 |
|  | Predicted 0 | 29 | 1 |
|  | Predicted 4 | 26 | 5 |
| Blood vs Infec | n=61 | Actual Blood | Actual Infec |
|  | Predicted Blood | 29 | 1 |
|  | Predicted Infec | 28 | 3 |
| Blood vs Metab | n=60 | Actual Blood | Actual Metab |
|  | Predicted Blood | 30 | 0 |
|  | Predicted Metab | 28 | 2 |
| Infec vs Metab | n=22 | Actual Infec | Actual Metab |
|  | Predicted Infec | 9 | 2 |
|  | Predicted Metab | 6 | 5 |

Predicted are the predictions from the model whereas actual refers to the true test labels.

# Test runs for Blood grade 0 vs grade 3

Table S5: Test runs for Blood grade 0 vs grade 3 model.

| Test run | AUC | Sensitivity  Rate | Specificity Rate |
| --- | --- | --- | --- |
| 1 | 1 | 0.80952381 | 0.85714286 |
| 2 | 1 | 0.81481481 | 0.875 |
| 3 | 1 | 0.78472222 | 0.875 |
| 4 | 1 | 0.82407407 | 0.875 |
| 5 | 1 | 0.7191358 | 0.91666667 |
| 6 | 1 | 0.81481481 | 0.85714286 |
| 7 | 1 | 0.66798942 | 0.92857143 |
| 8 | 1 | 0.83744856 | 0.88888889 |
| 9 | 1 | 0.75185185 | 0.9 |
| 10 | 1 | 0.82510288 | 0.88888889 |
| 11 | 1 | 0.79423868 | 0.88888889 |
| 12 | 1 | 0.70791246 | 0.95454545 |
| 13 | 1 | 0.68930041 | 0.94444444 |
| 14 | 1 | 0.75084175 | 0.90909091 |
| 15 | 1 | 0.70811287 | 0.95238095 |
| 16 | 1 | 0.79259259 | 0.9 |
| 17 | 1 | 0.83148148 | 0.9 |
| 18 | 1 | 0.79320988 | 0.91666667 |
| 19 | 1 | 0.82638889 | 0.875 |
| 20 | 1 | 0.77314815 | 0.875 |
| 21 | 1 | 0.80925926 | 0.9 |
| 22 | 1 | 0.74189815 | 0.9375 |
| 23 | 1 | 0.64484127 | 0.96428571 |
| 24 | 1 | 0.77160494 | 0.83333333 |
| 25 | 1 | 0.7962963 | 0.83333333 |
| 26 | 1 | 0.79365079 | 0.85714286 |
| 27 | 1 | 0.77513228 | 0.85714286 |
| 28 | 1 | 0.7962963 | 0.83333333 |
| 29 | 1 | 0.69312169 | 0.92857143 |
| 30 | 1 | 0.81944444 | 0.875 |
| 31 | 1 | 0.76190476 | 0.85714286 |
| 32 | 1 | 0.8127572 | 0.88888889 |
| 33 | 1 | 0.78009259 | 0.875 |
| 34 | 1 | 0.79320988 | 0.83333333 |
| 35 | 1 | 0.75396825 | 0.92857143 |
| 36 | 1 | 0.76666667 | 0.8 |
| 37 | 1 | 0.76430976 | 0.90909091 |
| 38 | 1 | 0.68634259 | 0.9375 |
| 39 | 1 | 0.79423868 | 0.88888889 |
| 40 | 1 | 0.7962963 | 0.85714286 |
| 41 | 1 | 0.78935185 | 0.875 |
| 42 | 1 | 0.82175926 | 0.875 |
| 43 | 1 | 0.7962963 | 0.83333333 |
| 44 | 1 | 0.76790123 | 0.93333333 |
| 45 | 1 | 0.77777778 | 0.83333333 |
| 46 | 1 | 0.79012346 | 0.88888889 |
| 47 | 1 | 0.78787879 | 0.90909091 |
| 48 | 1 | 0.78125 | 0.9375 |
| 49 | 1 | 0.81481481 | 0.9 |
| 50 | 1 | 0.80864198 | 0.83333333 |
| 51 | 1 | 0.78306878 | 0.85714286 |
| 52 | 1 | 0.76748971 | 0.88888889 |
| 53 | 1 | 0.81069959 | 0.88888889 |
| 54 | 1 | 0.72592593 | 0.93333333 |
| 55 | 1 | 0.73905724 | 0.90909091 |
| 56 | 1 | 0.79012346 | 0.83333333 |
| 57 | 1 | 0.73400673 | 0.90909091 |
| 58 | 1 | 0.78858025 | 0.91666667 |
| 59 | 1 | 0.79124579 | 0.90909091 |
| 60 | 1 | 0.74537037 | 0.875 |
| 61 | 1 | 0.75810185 | 0.9375 |
| 62 | 1 | 0.71759259 | 0.91666667 |
| 63 | 1 | 0.71111111 | 0.93333333 |
| 64 | 1 | 0.80555556 | 0.875 |
| 65 | 1 | 0.78518519 | 0.9 |
| 66 | 1 | 0.80808081 | 0.90909091 |
| 67 | 1 | 0.77366255 | 0.88888889 |
| 68 | 1 | 0.77609428 | 0.90909091 |
| 69 | 1 | 0.76234568 | 0.91666667 |
| 70 | 1 | 0.74074074 | 0.92307692 |
| 71 | 1 | 0.80092593 | 0.875 |
| 72 | 1 | 0.73580247 | 0.93333333 |
| 73 | 1 | 0.7962963 | 0.88888889 |
| 74 | 1 | 0.75231481 | 0.9375 |
| 75 | 1 | 0.79835391 | 0.88888889 |
| 76 | 1 | 0.76851852 | 0.875 |
| 77 | 1 | 0.6920078 | 0.94736842 |
| 78 | 1 | 0.84045584 | 0.92307692 |
| 79 | 1 | 0.72098765 | 0.93333333 |
| 80 | 1 | 0.7654321 | 0.94444444 |
| 81 | 1 | 0.79938272 | 0.91666667 |
| 82 | 1 | 0.75925926 | 0.9 |
| 83 | 1 | 0.79012346 | 0.88888889 |
| 84 | 1 | 0.80687831 | 0.85714286 |
| 85 | 1 | 0.77901235 | 0.93333333 |
| 86 | 1 | 0.80864198 | 0.83333333 |
| 87 | 1 | 0.78649237 | 0.94117647 |
| 88 | 1 | 0.70833333 | 0.9375 |
| 89 | 1 | 0.81712963 | 0.875 |
| 90 | 1 | 0.77946128 | 0.90909091 |
| 91 | 1 | 0.80324074 | 0.875 |
| 92 | 1 | 0.79059829 | 0.92307692 |
| 93 | 1 | 0.76430976 | 0.90909091 |
| 94 | 1 | 0.69753086 | 0.95238095 |
| 95 | 1 | 0.75810185 | 0.9375 |
| 96 | 1 | 0.76851852 | 0.875 |
| 97 | 1 | 0.79861111 | 0.875 |
| 98 | 1 | 0.80041152 | 0.88888889 |
| 99 | 1 | 0.7977208 | 0.92307692 |
| 100 | 1 | 0.79320988 | 0.83333333 |

# Test runs for Blood grade 0 vs grade 4

Table S6: Test runs for Blood grade 0 vs grade 4 model.

| Test run | AUC | Sensitivity  Rate | Specificity Rate |
| --- | --- | --- | --- |
| 1 | 0.70855438 | 0.59770115 | 0.60897436 |
| 2 | 0.68335544 | 0.58589624 | 0.59580735 |
| 3 | 0.70888594 | 0.59785648 | 0.60914761 |
| 4 | 0.69098143 | 0.58946878 | 0.5997921 |
| 5 | 0.69263926 | 0.59024542 | 0.60065835 |
| 6 | 0.67241379 | 0.58077043 | 0.59009009 |
| 7 | 0.71286472 | 0.59972041 | 0.61122661 |
| 8 | 0.68037135 | 0.58449829 | 0.59424809 |
| 9 | 0.68335544 | 0.58589624 | 0.59580735 |
| 10 | 0.70159151 | 0.59443927 | 0.60533611 |
| 11 | 0.69263926 | 0.59024542 | 0.60065835 |
| 12 | 0.7301061 | 0.60779745 | 0.62023562 |
| 13 | 0.72214854 | 0.60406959 | 0.61607762 |
| 14 | 0.6780504 | 0.583411 | 0.59303534 |
| 15 | 0.6979443 | 0.59273066 | 0.60343035 |
| 16 | 0.67937666 | 0.58403231 | 0.59372834 |
| 17 | 0.71949602 | 0.60282696 | 0.61469161 |
| 18 | 0.68136605 | 0.58496427 | 0.59476784 |
| 19 | 0.69927056 | 0.59335197 | 0.60412335 |
| 20 | 0.71253316 | 0.59956508 | 0.61105336 |
| 21 | 0.75928382 | 0.62146629 | 0.63548164 |
| 22 | 0.70623342 | 0.59661386 | 0.60776161 |
| 23 | 0.68335544 | 0.58589624 | 0.59580735 |
| 24 | 0.68070292 | 0.58465362 | 0.59442134 |
| 25 | 0.68037135 | 0.58449829 | 0.59424809 |
| 26 | 0.73507958 | 0.61012737 | 0.62283437 |
| 27 | 0.68037135 | 0.58449829 | 0.59424809 |
| 28 | 0.70092838 | 0.59412861 | 0.6049896 |
| 29 | 0.67374005 | 0.58139174 | 0.59078309 |
| 30 | 0.68965517 | 0.58884747 | 0.5990991 |
| 31 | 0.67771883 | 0.58325567 | 0.59286209 |
| 32 | 0.70689655 | 0.59692451 | 0.60810811 |
| 33 | 0.67606101 | 0.58247903 | 0.59199584 |
| 34 | 0.67572944 | 0.5823237 | 0.59182259 |
| 35 | 0.71054377 | 0.59863312 | 0.61001386 |
| 36 | 0.70258621 | 0.59490525 | 0.60585586 |
| 37 | 0.68733422 | 0.58776017 | 0.59788635 |
| 38 | 0.70590186 | 0.59645853 | 0.60758836 |
| 39 | 0.69330239 | 0.59055607 | 0.60100485 |
| 40 | 0.67639257 | 0.58263436 | 0.59216909 |
| 41 | 0.6903183 | 0.58915812 | 0.5994456 |
| 42 | 0.71087533 | 0.59878844 | 0.61018711 |
| 43 | 0.69263926 | 0.59024542 | 0.60065835 |
| 44 | 0.70092838 | 0.59412861 | 0.6049896 |
| 45 | 0.72679045 | 0.60624418 | 0.61850312 |
| 46 | 0.71319629 | 0.59987574 | 0.61139986 |
| 47 | 0.71982759 | 0.60298229 | 0.61486486 |
| 48 | 0.67108753 | 0.58014911 | 0.58939709 |
| 49 | 0.67241379 | 0.58077043 | 0.59009009 |
| 50 | 0.68567639 | 0.58698354 | 0.5970201 |
| 51 | 0.67572944 | 0.5823237 | 0.59182259 |
| 52 | 0.7367374 | 0.61090401 | 0.62370062 |
| 53 | 0.71949602 | 0.60282696 | 0.61469161 |
| 54 | 0.70490716 | 0.59599254 | 0.60706861 |
| 55 | 0.72480106 | 0.60531221 | 0.61746362 |
| 56 | 0.70324934 | 0.59521591 | 0.60620236 |
| 57 | 0.70258621 | 0.59490525 | 0.60585586 |
| 58 | 0.70391247 | 0.59552656 | 0.60654886 |
| 59 | 0.73706897 | 0.61105934 | 0.62387387 |
| 60 | 0.69860743 | 0.59304132 | 0.60377685 |
| 61 | 0.70988064 | 0.59832246 | 0.60966736 |
| 62 | 0.69562334 | 0.59164337 | 0.6022176 |
| 63 | 0.72115385 | 0.6036036 | 0.61555787 |
| 64 | 0.70822281 | 0.59754582 | 0.60880111 |
| 65 | 0.70921751 | 0.5980118 | 0.60932086 |
| 66 | 0.7178382 | 0.60205033 | 0.61382536 |
| 67 | 0.67639257 | 0.58263436 | 0.59216909 |
| 68 | 0.71916446 | 0.60267164 | 0.61451836 |
| 69 | 0.70523873 | 0.59614787 | 0.60724186 |
| 70 | 0.68401857 | 0.5862069 | 0.59615385 |
| 71 | 0.69993369 | 0.59366263 | 0.60446985 |
| 72 | 0.68932361 | 0.58869214 | 0.59892585 |
| 73 | 0.68401857 | 0.5862069 | 0.59615385 |
| 74 | 0.67440318 | 0.58170239 | 0.59112959 |
| 75 | 0.69263926 | 0.59024542 | 0.60065835 |
| 76 | 0.71982759 | 0.60298229 | 0.61486486 |
| 77 | 0.70988064 | 0.59832246 | 0.60966736 |
| 78 | 0.67374005 | 0.58139174 | 0.59078309 |
| 79 | 0.69728117 | 0.59242001 | 0.60308385 |
| 80 | 0.69927056 | 0.59335197 | 0.60412335 |
| 81 | 0.6903183 | 0.58915812 | 0.5994456 |
| 82 | 0.683687 | 0.58605157 | 0.5959806 |
| 83 | 0.70159151 | 0.59443927 | 0.60533611 |
| 84 | 0.683687 | 0.58605157 | 0.5959806 |
| 85 | 0.72679045 | 0.60624418 | 0.61850312 |
| 86 | 0.70291777 | 0.59506058 | 0.60602911 |
| 87 | 0.68899204 | 0.58853681 | 0.5987526 |
| 88 | 0.69893899 | 0.59319664 | 0.6039501 |
| 89 | 0.71916446 | 0.60267164 | 0.61451836 |
| 90 | 0.67374005 | 0.58139174 | 0.59078309 |
| 91 | 0.71419098 | 0.60034172 | 0.61191961 |
| 92 | 0.68965517 | 0.58884747 | 0.5990991 |
| 93 | 0.7045756 | 0.59583722 | 0.60689536 |
| 94 | 0.70921751 | 0.5980118 | 0.60932086 |
| 95 | 0.70291777 | 0.59506058 | 0.60602911 |
| 96 | 0.70988064 | 0.59832246 | 0.60966736 |
| 97 | 0.70192308 | 0.59459459 | 0.60550936 |
| 98 | 0.70258621 | 0.59490525 | 0.60585586 |
| 99 | 0.70391247 | 0.59552656 | 0.60654886 |
| 100 | 0.73375332 | 0.60950606 | 0.62214137 |

# Test runs for Blood grade 3 vs grade 4

Table S7: Test runs for Blood grade 3 vs grade 4 model.

| Test run | AUC | Sensitivity  Rate | Specificity Rate |
| --- | --- | --- | --- |
| 1 | 0.62743802 | 0.56314496 | 0.56314496 |
| 2 | 0.65256198 | 0.57559378 | 0.57559378 |
| 3 | 0.67338843 | 0.58591319 | 0.58591319 |
| 4 | 0.6707438 | 0.58460278 | 0.58460278 |
| 5 | 0.64231405 | 0.57051597 | 0.57051597 |
| 6 | 0.63933884 | 0.56904177 | 0.56904177 |
| 7 | 0.65520661 | 0.57690418 | 0.57690418 |
| 8 | 0.65553719 | 0.57706798 | 0.57706798 |
| 9 | 0.64528926 | 0.57199017 | 0.57199017 |
| 10 | 0.64132231 | 0.57002457 | 0.57002457 |
| 11 | 0.69256198 | 0.5954136 | 0.5954136 |
| 12 | 0.65421488 | 0.57641278 | 0.57641278 |
| 13 | 0.65586777 | 0.57723178 | 0.57723178 |
| 14 | 0.63735537 | 0.56805897 | 0.56805897 |
| 15 | 0.6538843 | 0.57624898 | 0.57624898 |
| 16 | 0.63008264 | 0.56445536 | 0.56445536 |
| 17 | 0.63702479 | 0.56789517 | 0.56789517 |
| 18 | 0.63669421 | 0.56773137 | 0.56773137 |
| 19 | 0.64958678 | 0.57411957 | 0.57411957 |
| 20 | 0.6707438 | 0.58460278 | 0.58460278 |
| 21 | 0.63305785 | 0.56592957 | 0.56592957 |
| 22 | 0.67636364 | 0.58738739 | 0.58738739 |
| 23 | 0.63438017 | 0.56658477 | 0.56658477 |
| 24 | 0.71404959 | 0.60606061 | 0.60606061 |
| 25 | 0.65057851 | 0.57461097 | 0.57461097 |
| 26 | 0.63338843 | 0.56609337 | 0.56609337 |
| 27 | 0.63669421 | 0.56773137 | 0.56773137 |
| 28 | 0.63140496 | 0.56511057 | 0.56511057 |
| 29 | 0.63966942 | 0.56920557 | 0.56920557 |
| 30 | 0.63173554 | 0.56527437 | 0.56527437 |
| 31 | 0.63107438 | 0.56494676 | 0.56494676 |
| 32 | 0.63206612 | 0.56543817 | 0.56543817 |
| 33 | 0.63140496 | 0.56511057 | 0.56511057 |
| 34 | 0.64595041 | 0.57231777 | 0.57231777 |
| 35 | 0.6568595 | 0.57772318 | 0.57772318 |
| 36 | 0.64958678 | 0.57411957 | 0.57411957 |
| 37 | 0.63834711 | 0.56855037 | 0.56855037 |
| 38 | 0.65355372 | 0.57608518 | 0.57608518 |
| 39 | 0.66446281 | 0.58149058 | 0.58149058 |
| 40 | 0.68396694 | 0.59115479 | 0.59115479 |
| 41 | 0.64132231 | 0.57002457 | 0.57002457 |
| 42 | 0.64330579 | 0.57100737 | 0.57100737 |
| 43 | 0.64396694 | 0.57133497 | 0.57133497 |
| 44 | 0.63471074 | 0.56674857 | 0.56674857 |
| 45 | 0.63206612 | 0.56543817 | 0.56543817 |
| 46 | 0.68033058 | 0.58935299 | 0.58935299 |
| 47 | 0.63272727 | 0.56576577 | 0.56576577 |
| 48 | 0.71173554 | 0.604914 | 0.604914 |
| 49 | 0.63603306 | 0.56740377 | 0.56740377 |
| 50 | 0.63570248 | 0.56723997 | 0.56723997 |
| 51 | 0.65652893 | 0.57755938 | 0.57755938 |
| 52 | 0.64628099 | 0.57248157 | 0.57248157 |
| 53 | 0.62280992 | 0.56085176 | 0.56085176 |
| 54 | 0.67603306 | 0.58722359 | 0.58722359 |
| 55 | 0.67239669 | 0.58542179 | 0.58542179 |
| 56 | 0.63801653 | 0.56838657 | 0.56838657 |
| 57 | 0.63966942 | 0.56920557 | 0.56920557 |
| 58 | 0.65024793 | 0.57444717 | 0.57444717 |
| 59 | 0.63305785 | 0.56592957 | 0.56592957 |
| 60 | 0.67966942 | 0.58902539 | 0.58902539 |
| 61 | 0.63140496 | 0.56511057 | 0.56511057 |
| 62 | 0.65619835 | 0.57739558 | 0.57739558 |
| 63 | 0.63834711 | 0.56855037 | 0.56855037 |
| 64 | 0.64033058 | 0.56953317 | 0.56953317 |
| 65 | 0.65322314 | 0.57592138 | 0.57592138 |
| 66 | 0.64760331 | 0.57313677 | 0.57313677 |
| 67 | 0.66644628 | 0.58247338 | 0.58247338 |
| 68 | 0.64429752 | 0.57149877 | 0.57149877 |
| 69 | 0.65983471 | 0.57919738 | 0.57919738 |
| 70 | 0.67669421 | 0.58755119 | 0.58755119 |
| 71 | 0.63438017 | 0.56658477 | 0.56658477 |
| 72 | 0.66512397 | 0.58181818 | 0.58181818 |
| 73 | 0.64561983 | 0.57215397 | 0.57215397 |
| 74 | 0.65322314 | 0.57592138 | 0.57592138 |
| 75 | 0.63966942 | 0.56920557 | 0.56920557 |
| 76 | 0.64958678 | 0.57411957 | 0.57411957 |
| 77 | 0.63206612 | 0.56543817 | 0.56543817 |
| 78 | 0.65520661 | 0.57690418 | 0.57690418 |
| 79 | 0.6661157 | 0.58230958 | 0.58230958 |
| 80 | 0.65057851 | 0.57461097 | 0.57461097 |
| 81 | 0.63768595 | 0.56822277 | 0.56822277 |
| 82 | 0.67867769 | 0.58853399 | 0.58853399 |
| 83 | 0.68727273 | 0.59279279 | 0.59279279 |
| 84 | 0.64958678 | 0.57411957 | 0.57411957 |
| 85 | 0.64363636 | 0.57117117 | 0.57117117 |
| 86 | 0.65454545 | 0.57657658 | 0.57657658 |
| 87 | 0.67371901 | 0.58607699 | 0.58607699 |
| 88 | 0.64561983 | 0.57215397 | 0.57215397 |
| 89 | 0.64495868 | 0.57182637 | 0.57182637 |
| 90 | 0.63900826 | 0.56887797 | 0.56887797 |
| 91 | 0.64628099 | 0.57248157 | 0.57248157 |
| 92 | 0.64198347 | 0.57035217 | 0.57035217 |
| 93 | 0.64 | 0.56936937 | 0.56936937 |
| 94 | 0.66578512 | 0.58214578 | 0.58214578 |
| 95 | 0.65785124 | 0.57821458 | 0.57821458 |
| 96 | 0.6677686 | 0.58312858 | 0.58312858 |
| 97 | 0.66545455 | 0.58198198 | 0.58198198 |
| 98 | 0.66644628 | 0.58247338 | 0.58247338 |
| 99 | 0.63702479 | 0.56789517 | 0.56789517 |
| 100 | 0.64826446 | 0.57346437 | 0.57346437 |

# Test runs for Infec grade 0 vs grade 3

Table S8: Test runs for Infec grade 3 vs grade 4 model.

| Test run | AUC | Sensitivity  Rate | Specificity Rate |
| --- | --- | --- | --- |
| 1 | 0.73365617 | 0.68629344 | 0.54420522 |
| 2 | 0.70581114 | 0.66409266 | 0.53893724 |
| 3 | 0.67917676 | 0.64285714 | 0.53389831 |
| 4 | 0.70460048 | 0.66312741 | 0.5387082 |
| 5 | 0.6598063 | 0.62741313 | 0.53023362 |
| 6 | 0.65617433 | 0.62451737 | 0.5295465 |
| 7 | 0.71307506 | 0.66988417 | 0.5403115 |
| 8 | 0.61501211 | 0.59169884 | 0.52175905 |
| 9 | 0.60774818 | 0.58590734 | 0.52038479 |
| 10 | 0.64527845 | 0.61583012 | 0.52748511 |
| 11 | 0.69491525 | 0.65540541 | 0.53687586 |
| 12 | 0.67917676 | 0.64285714 | 0.53389831 |
| 13 | 0.69975787 | 0.65926641 | 0.53779203 |
| 14 | 0.73365617 | 0.68629344 | 0.54420522 |
| 15 | 0.67917676 | 0.64285714 | 0.53389831 |
| 16 | 0.62590799 | 0.6003861 | 0.52382043 |
| 17 | 0.7433414 | 0.69401544 | 0.54603756 |
| 18 | 0.63438257 | 0.60714286 | 0.52542373 |
| 19 | 0.85108959 | 0.77992278 | 0.56642235 |
| 20 | 0.70702179 | 0.66505792 | 0.53916628 |
| 21 | 0.77845036 | 0.72200772 | 0.5526798 |
| 22 | 0.73244552 | 0.68532819 | 0.54397618 |
| 23 | 0.64769976 | 0.61776062 | 0.5279432 |
| 24 | 0.74818402 | 0.69787645 | 0.54695373 |
| 25 | 0.7433414 | 0.69401544 | 0.54603756 |
| 26 | 0.71549637 | 0.67181467 | 0.54076958 |
| 27 | 0.69975787 | 0.65926641 | 0.53779203 |
| 28 | 0.73849879 | 0.69015444 | 0.54512139 |
| 29 | 0.62227603 | 0.59749035 | 0.5231333 |
| 30 | 0.7094431 | 0.66698842 | 0.53962437 |
| 31 | 0.71065375 | 0.66795367 | 0.53985341 |
| 32 | 0.7433414 | 0.69401544 | 0.54603756 |
| 33 | 0.69007264 | 0.6515444 | 0.53595969 |
| 34 | 0.70581114 | 0.66409266 | 0.53893724 |
| 35 | 0.69854722 | 0.65830116 | 0.53756299 |
| 36 | 0.71670702 | 0.67277992 | 0.54099863 |
| 37 | 0.65859564 | 0.62644788 | 0.53000458 |
| 38 | 0.62590799 | 0.6003861 | 0.52382043 |
| 39 | 0.71912833 | 0.67471042 | 0.54145671 |
| 40 | 0.77360775 | 0.71814672 | 0.55176363 |
| 41 | 0.72639225 | 0.68050193 | 0.54283097 |
| 42 | 0.63801453 | 0.61003861 | 0.52611086 |
| 43 | 0.72881356 | 0.68243243 | 0.54328905 |
| 44 | 0.78329298 | 0.72586873 | 0.55359597 |
| 45 | 0.73002421 | 0.68339768 | 0.54351809 |
| 46 | 0.7409201 | 0.69208494 | 0.54557948 |
| 47 | 0.7409201 | 0.69208494 | 0.54557948 |
| 48 | 0.65859564 | 0.62644788 | 0.53000458 |
| 49 | 0.64043584 | 0.61196911 | 0.52656894 |
| 50 | 0.66828087 | 0.63416988 | 0.53183692 |
| 51 | 0.67917676 | 0.64285714 | 0.53389831 |
| 52 | 0.65254237 | 0.62162162 | 0.52885937 |
| 53 | 0.63680387 | 0.60907336 | 0.52588181 |
| 54 | 0.60653753 | 0.58494208 | 0.52015575 |
| 55 | 0.65496368 | 0.62355212 | 0.52931745 |
| 56 | 0.68280872 | 0.6457529 | 0.53458543 |
| 57 | 0.63438257 | 0.60714286 | 0.52542373 |
| 58 | 0.74697337 | 0.6969112 | 0.54672469 |
| 59 | 0.70217918 | 0.66119691 | 0.53825011 |
| 60 | 0.66707022 | 0.63320463 | 0.53160788 |
| 61 | 0.69975787 | 0.65926641 | 0.53779203 |
| 62 | 0.70096852 | 0.66023166 | 0.53802107 |
| 63 | 0.66464891 | 0.63127413 | 0.53114979 |
| 64 | 0.64164649 | 0.61293436 | 0.52679798 |
| 65 | 0.71307506 | 0.66988417 | 0.5403115 |
| 66 | 0.66949153 | 0.63513514 | 0.53206596 |
| 67 | 0.80266344 | 0.74131274 | 0.55726065 |
| 68 | 0.73123487 | 0.68436293 | 0.54374714 |
| 69 | 0.66585956 | 0.63223938 | 0.53137884 |
| 70 | 0.74576271 | 0.69594595 | 0.54649565 |
| 71 | 0.76876513 | 0.71428571 | 0.55084746 |
| 72 | 0.64769976 | 0.61776062 | 0.5279432 |
| 73 | 0.73970944 | 0.69111969 | 0.54535044 |
| 74 | 0.71307506 | 0.66988417 | 0.5403115 |
| 75 | 0.71549637 | 0.67181467 | 0.54076958 |
| 76 | 0.73607748 | 0.68822394 | 0.54466331 |
| 77 | 0.75181598 | 0.7007722 | 0.54764086 |
| 78 | 0.79903148 | 0.73841699 | 0.55657352 |
| 79 | 0.73002421 | 0.68339768 | 0.54351809 |
| 80 | 0.81234867 | 0.74903475 | 0.55909299 |
| 81 | 0.68765133 | 0.6496139 | 0.5355016 |
| 82 | 0.68765133 | 0.6496139 | 0.5355016 |
| 83 | 0.64891041 | 0.61872587 | 0.52817224 |
| 84 | 0.78087167 | 0.72393822 | 0.55313788 |
| 85 | 0.6937046 | 0.65444015 | 0.53664682 |
| 86 | 0.76029056 | 0.70752896 | 0.54924416 |
| 87 | 0.65375303 | 0.62258687 | 0.52908841 |
| 88 | 0.61743341 | 0.59362934 | 0.52221713 |
| 89 | 0.82929782 | 0.76254826 | 0.56229959 |
| 90 | 0.8062954 | 0.74420849 | 0.55794778 |
| 91 | 0.7094431 | 0.66698842 | 0.53962437 |
| 92 | 0.64164649 | 0.61293436 | 0.52679798 |
| 93 | 0.70823245 | 0.66602317 | 0.53939533 |
| 94 | 0.69733656 | 0.65733591 | 0.53733394 |
| 95 | 0.73728814 | 0.68918919 | 0.54489235 |
| 96 | 0.60895884 | 0.58687259 | 0.52061383 |
| 97 | 0.65617433 | 0.62451737 | 0.5295465 |
| 98 | 0.61501211 | 0.59169884 | 0.52175905 |
| 99 | 0.70581114 | 0.66409266 | 0.53893724 |
| 100 | 0.72639225 | 0.68050193 | 0.54283097 |

# Test runs for Infec grade 0 vs grade 4

Table S9: Test runs for Infec grade 0 vs grade 4 model.

| Test run | AUC | Sensitivity  Rate | Specificity Rate |
| --- | --- | --- | --- |
| 1 | 0.66022727 | 0.59375 | 0.58181818 |
| 2 | 0.71818182 | 0.6875 | 0.52727273 |
| 3 | 0.73409091 | 0.69642857 | 0.53593074 |
| 4 | 0.73181818 | 0.69921875 | 0.52897727 |
| 5 | 0.63409091 | 0.61523438 | 0.51676136 |
| 6 | 0.70681818 | 0.67773438 | 0.52585227 |
| 7 | 0.72954545 | 0.69726563 | 0.52869318 |
| 8 | 0.65 | 0.62890625 | 0.51875 |
| 9 | 0.65681818 | 0.63476563 | 0.51960227 |
| 10 | 0.70909091 | 0.6796875 | 0.52613636 |
| 11 | 0.73181818 | 0.69921875 | 0.52897727 |
| 12 | 0.71818182 | 0.6875 | 0.52727273 |
| 13 | 0.67727273 | 0.64880952 | 0.52554113 |
| 14 | 0.72272727 | 0.69140625 | 0.52784091 |
| 15 | 0.68636364 | 0.66015625 | 0.52329545 |
| 16 | 0.68636364 | 0.6547619 | 0.53044733 |
| 17 | 0.74545455 | 0.7109375 | 0.53068182 |
| 18 | 0.70909091 | 0.6796875 | 0.52613636 |
| 19 | 0.74318182 | 0.70898438 | 0.53039773 |
| 20 | 0.71363636 | 0.68359375 | 0.52670455 |
| 21 | 0.75681818 | 0.72070313 | 0.53210227 |
| 22 | 0.78181818 | 0.7421875 | 0.53522727 |
| 23 | 0.75 | 0.71484375 | 0.53125 |
| 24 | 0.74545455 | 0.70833333 | 0.53477633 |
| 25 | 0.63181818 | 0.61328125 | 0.51647727 |
| 26 | 0.66818182 | 0.64880952 | 0.51688312 |
| 27 | 0.72727273 | 0.6953125 | 0.52840909 |
| 28 | 0.73409091 | 0.6984127 | 0.53333333 |
| 29 | 0.63181818 | 0.61328125 | 0.51647727 |
| 30 | 0.70909091 | 0.67460317 | 0.53333333 |
| 31 | 0.69545455 | 0.66796875 | 0.52443182 |
| 32 | 0.81590909 | 0.77148438 | 0.53948864 |
| 33 | 0.72045455 | 0.68945313 | 0.52755682 |
| 34 | 0.65681818 | 0.63293651 | 0.52582973 |
| 35 | 0.74772727 | 0.70833333 | 0.53535354 |
| 36 | 0.65454545 | 0.64285714 | 0.51226551 |
| 37 | 0.74090909 | 0.70703125 | 0.53011364 |
| 38 | 0.64545455 | 0.625 | 0.51818182 |
| 39 | 0.72954545 | 0.69444444 | 0.53275613 |
| 40 | 0.68636364 | 0.66015625 | 0.52329545 |
| 41 | 0.7 | 0.671875 | 0.525 |
| 42 | 0.69545455 | 0.66796875 | 0.52443182 |
| 43 | 0.75227273 | 0.71679688 | 0.53153409 |
| 44 | 0.72272727 | 0.69140625 | 0.52784091 |
| 45 | 0.69545455 | 0.66796875 | 0.52443182 |
| 46 | 0.67954545 | 0.65429688 | 0.52244318 |
| 47 | 0.69318182 | 0.66601563 | 0.52414773 |
| 48 | 0.68409091 | 0.65820313 | 0.52301136 |
| 49 | 0.64772727 | 0.625 | 0.52323232 |
| 50 | 0.67954545 | 0.65429688 | 0.52244318 |
| 51 | 0.73863636 | 0.70507813 | 0.52982955 |
| 52 | 0.80681818 | 0.76367188 | 0.53835227 |
| 53 | 0.67045455 | 0.64484127 | 0.52438672 |
| 54 | 0.75454545 | 0.71875 | 0.53181818 |
| 55 | 0.75681818 | 0.71626984 | 0.53852814 |
| 56 | 0.71818182 | 0.6875 | 0.52727273 |
| 57 | 0.71818182 | 0.6875 | 0.52727273 |
| 58 | 0.64545455 | 0.625 | 0.51818182 |
| 59 | 0.64545455 | 0.625 | 0.51818182 |
| 60 | 0.63636364 | 0.6171875 | 0.51704545 |
| 61 | 0.73181818 | 0.69444444 | 0.53708514 |
| 62 | 0.67727273 | 0.65234375 | 0.52215909 |
| 63 | 0.73181818 | 0.69642857 | 0.53333333 |
| 64 | 0.79318182 | 0.75195313 | 0.53664773 |
| 65 | 0.70909091 | 0.6796875 | 0.52613636 |
| 66 | 0.63636364 | 0.61111111 | 0.52352092 |
| 67 | 0.64545455 | 0.62301587 | 0.52409812 |
| 68 | 0.65 | 0.62890625 | 0.51875 |
| 69 | 0.70227273 | 0.67460317 | 0.52323232 |
| 70 | 0.72727273 | 0.6953125 | 0.52840909 |
| 71 | 0.69318182 | 0.66601563 | 0.52414773 |
| 72 | 0.64090909 | 0.62109375 | 0.51761364 |
| 73 | 0.69772727 | 0.66666667 | 0.53015873 |
| 74 | 0.76136364 | 0.72460938 | 0.53267045 |
| 75 | 0.79772727 | 0.75585938 | 0.53721591 |
| 76 | 0.65909091 | 0.63293651 | 0.52380952 |
| 77 | 0.66590909 | 0.64257813 | 0.52073864 |
| 78 | 0.73863636 | 0.70507813 | 0.52982955 |
| 79 | 0.63863636 | 0.61914063 | 0.51732955 |
| 80 | 0.65454545 | 0.6328125 | 0.51931818 |
| 81 | 0.675 | 0.65039063 | 0.521875 |
| 82 | 0.69772727 | 0.66666667 | 0.52611833 |
| 83 | 0.64772727 | 0.62695313 | 0.51846591 |
| 84 | 0.65681818 | 0.62896825 | 0.52669553 |
| 85 | 0.65909091 | 0.63671875 | 0.51988636 |
| 86 | 0.79772727 | 0.75585938 | 0.53721591 |
| 87 | 0.7 | 0.671875 | 0.525 |
| 88 | 0.72727273 | 0.6953125 | 0.52840909 |
| 89 | 0.63863636 | 0.61914063 | 0.51732955 |
| 90 | 0.70454545 | 0.67063492 | 0.52987013 |
| 91 | 0.71818182 | 0.68253968 | 0.53535354 |
| 92 | 0.675 | 0.65039063 | 0.521875 |
| 93 | 0.66136364 | 0.63867188 | 0.52017045 |
| 94 | 0.725 | 0.69335938 | 0.528125 |
| 95 | 0.70227273 | 0.67261905 | 0.52467532 |
| 96 | 0.73181818 | 0.69444444 | 0.53708514 |
| 97 | 0.66590909 | 0.64257813 | 0.52073864 |
| 98 | 0.63636364 | 0.6171875 | 0.51704545 |
| 99 | 0.66363636 | 0.63492063 | 0.52756133 |
| 100 | 0.65227273 | 0.64087302 | 0.51197691 |

# Test runs for Infec grade 0 vs grade 5

Table S10: Test runs for Infec grade 0 vs grade 5 model.

| Test run | AUC | Sensitivity  Rate | Specificity Rate |
| --- | --- | --- | --- |
| 1 | 0.72321429 | 0.75892857 | 0.4375 |
| 2 | 0.72991071 | 0.6875 | 0.54017857 |
| 3 | 0.71205357 | 0.63888889 | 0.54960317 |
| 4 | 0.71651786 | 0.71875 | 0.47544643 |
| 5 | 0.70535714 | 0.69117647 | 0.51365546 |
| 6 | 0.70535714 | 0.69444444 | 0.46031746 |
| 7 | 0.69866071 | 0.76785714 | 0.44260204 |
| 8 | 0.72991071 | 0.69444444 | 0.50396825 |
| 9 | 0.74776786 | 0.75 | 0.50698758 |
| 10 | 0.64285714 | 0.61904762 | 0.49234694 |
| 11 | 0.71651786 | 0.75 | 0.44444444 |
| 12 | 0.64285714 | 0.625 | 0.51897321 |
| 13 | 0.72767857 | 0.77777778 | 0.43452381 |
| 14 | 0.68526786 | 0.73611111 | 0.42559524 |
| 15 | 0.6875 | 0.63888889 | 0.51984127 |
| 16 | 0.71875 | 0.65625 | 0.58482143 |
| 17 | 0.70758929 | 0.72222222 | 0.46428571 |
| 18 | 0.65848214 | 0.69444444 | 0.45833333 |
| 19 | 0.66517857 | 0.57894737 | 0.59398496 |
| 20 | 0.67857143 | 0.70833333 | 0.45238095 |
| 21 | 0.70535714 | 0.67105263 | 0.4962406 |
| 22 | 0.70535714 | 0.59722222 | 0.56746032 |
| 23 | 0.74330357 | 0.71296296 | 0.49669312 |
| 24 | 0.70089286 | 0.72368421 | 0.45676692 |
| 25 | 0.66741071 | 0.63333333 | 0.52261905 |
| 26 | 0.69642857 | 0.64583333 | 0.54613095 |
| 27 | 0.79910714 | 0.81818182 | 0.43506494 |
| 28 | 0.78125 | 0.828125 | 0.44977679 |
| 29 | 0.66741071 | 0.70833333 | 0.44642857 |
| 30 | 0.68973214 | 0.69444444 | 0.46825397 |
| 31 | 0.71428571 | 0.60416667 | 0.57589286 |
| 32 | 0.734375 | 0.70833333 | 0.47619048 |
| 33 | 0.69642857 | 0.6875 | 0.51934524 |
| 34 | 0.73660714 | 0.72222222 | 0.45634921 |
| 35 | 0.72321429 | 0.73333333 | 0.43214286 |
| 36 | 0.68303571 | 0.625 | 0.56051587 |
| 37 | 0.68080357 | 0.68518519 | 0.46560847 |
| 38 | 0.671875 | 0.7 | 0.44642857 |
| 39 | 0.71205357 | 0.66666667 | 0.52777778 |
| 40 | 0.71205357 | 0.65625 | 0.53236607 |
| 41 | 0.71651786 | 0.69444444 | 0.48015873 |
| 42 | 0.70758929 | 0.72058824 | 0.45903361 |
| 43 | 0.67410714 | 0.65 | 0.47857143 |
| 44 | 0.65848214 | 0.66666667 | 0.46130952 |
| 45 | 0.71651786 | 0.67857143 | 0.50255102 |
| 46 | 0.64508929 | 0.5 | 0.62053571 |
| 47 | 0.68973214 | 0.75 | 0.40119048 |
| 48 | 0.69419643 | 0.65 | 0.49285714 |
| 49 | 0.78125 | 0.68333333 | 0.5797619 |
| 50 | 0.67857143 | 0.66176471 | 0.48529412 |
| 51 | 0.68526786 | 0.68181818 | 0.50324675 |
| 52 | 0.73214286 | 0.72222222 | 0.46230159 |
| 53 | 0.70758929 | 0.70833333 | 0.4672619 |
| 54 | 0.71651786 | 0.734375 | 0.48102679 |
| 55 | 0.68303571 | 0.72222222 | 0.41071429 |
| 56 | 0.70982143 | 0.75 | 0.43551587 |
| 57 | 0.68303571 | 0.66346154 | 0.51236264 |
| 58 | 0.72767857 | 0.671875 | 0.53125 |
| 59 | 0.65848214 | 0.69444444 | 0.44444444 |
| 60 | 0.671875 | 0.66666667 | 0.46626984 |
| 61 | 0.72544643 | 0.78846154 | 0.43406593 |
| 62 | 0.72544643 | 0.72619048 | 0.48894558 |
| 63 | 0.70982143 | 0.69444444 | 0.5 |
| 64 | 0.78794643 | 0.80555556 | 0.43055556 |
| 65 | 0.66964286 | 0.69444444 | 0.43452381 |
| 66 | 0.72321429 | 0.65909091 | 0.51461039 |
| 67 | 0.75 | 0.78125 | 0.47544643 |
| 68 | 0.67633929 | 0.63888889 | 0.50396825 |
| 69 | 0.796875 | 0.7 | 0.53928571 |
| 70 | 0.72991071 | 0.75 | 0.46703297 |
| 71 | 0.76339286 | 0.72916667 | 0.48958333 |
| 72 | 0.72544643 | 0.65740741 | 0.54298942 |
| 73 | 0.76339286 | 0.68181818 | 0.53409091 |
| 74 | 0.734375 | 0.7 | 0.525 |
| 75 | 0.72767857 | 0.70833333 | 0.50744048 |
| 76 | 0.73660714 | 0.75 | 0.44345238 |
| 77 | 0.67633929 | 0.734375 | 0.42633929 |
| 78 | 0.75892857 | 0.77586207 | 0.49692118 |
| 79 | 0.65625 | 0.71212121 | 0.46049784 |
| 80 | 0.72767857 | 0.671875 | 0.53459821 |
| 81 | 0.796875 | 0.75 | 0.49285714 |
| 82 | 0.67410714 | 0.66666667 | 0.46825397 |
| 83 | 0.67410714 | 0.70238095 | 0.46853741 |
| 84 | 0.671875 | 0.70454545 | 0.46103896 |
| 85 | 0.67410714 | 0.63888889 | 0.50396825 |
| 86 | 0.71651786 | 0.6875 | 0.50111607 |
| 87 | 0.70089286 | 0.68269231 | 0.49381868 |
| 88 | 0.65401786 | 0.6 | 0.53928571 |
| 89 | 0.72544643 | 0.72916667 | 0.44494048 |
| 90 | 0.71428571 | 0.6875 | 0.50446429 |
| 91 | 0.73660714 | 0.6875 | 0.53794643 |
| 92 | 0.66517857 | 0.69444444 | 0.46329365 |
| 93 | 0.71875 | 0.675 | 0.53571429 |
| 94 | 0.70982143 | 0.68181818 | 0.49512987 |
| 95 | 0.70982143 | 0.70833333 | 0.48809524 |
| 96 | 0.71875 | 0.72413793 | 0.48891626 |
| 97 | 0.66741071 | 0.66666667 | 0.51666667 |
| 98 | 0.671875 | 0.63888889 | 0.47420635 |
| 99 | 0.70089286 | 0.77777778 | 0.42460317 |
| 100 | 0.703125 | 0.6875 | 0.50111607 |

# Test runs for Infec grade 3 vs grade 4

Table S11: Test runs for Infec grade 3 vs grade 4 model.

| Test run | AUC | Sensitivity  Rate | Specificity Rate |
| --- | --- | --- | --- |
| 1 | 0.7 | 0.63636364 | 0.55454545 |
| 2 | 0.7 | 0.63636364 | 0.55454545 |
| 3 | 0.72222222 | 0.65151515 | 0.56060606 |
| 4 | 0.74444444 | 0.66666667 | 0.56666667 |
| 5 | 0.71111111 | 0.64393939 | 0.55757576 |
| 6 | 0.64444444 | 0.59848485 | 0.53939394 |
| 7 | 0.72222222 | 0.65151515 | 0.56060606 |
| 8 | 0.67777778 | 0.62121212 | 0.54848485 |
| 9 | 0.71111111 | 0.64393939 | 0.55757576 |
| 10 | 0.77777778 | 0.68939394 | 0.57575758 |
| 11 | 0.7 | 0.63636364 | 0.55454545 |
| 12 | 0.74444444 | 0.66666667 | 0.56666667 |
| 13 | 0.71111111 | 0.64393939 | 0.55757576 |
| 14 | 0.71111111 | 0.64393939 | 0.55757576 |
| 15 | 0.67777778 | 0.62121212 | 0.54848485 |
| 16 | 0.76666667 | 0.68181818 | 0.57272727 |
| 17 | 0.7 | 0.63636364 | 0.55454545 |
| 18 | 0.66666667 | 0.61363636 | 0.54545455 |
| 19 | 0.74444444 | 0.66666667 | 0.56666667 |
| 20 | 0.77777778 | 0.68939394 | 0.57575758 |
| 21 | 0.67777778 | 0.62121212 | 0.54848485 |
| 22 | 0.66666667 | 0.61363636 | 0.54545455 |
| 23 | 0.78888889 | 0.6969697 | 0.57878788 |
| 24 | 0.77777778 | 0.68939394 | 0.57575758 |
| 25 | 0.73333333 | 0.65909091 | 0.56363636 |
| 26 | 0.75555556 | 0.67424242 | 0.56969697 |
| 27 | 0.7 | 0.63636364 | 0.55454545 |
| 28 | 0.72222222 | 0.65151515 | 0.56060606 |
| 29 | 0.81111111 | 0.71212121 | 0.58484848 |
| 30 | 0.74444444 | 0.66666667 | 0.56666667 |
| 31 | 0.73333333 | 0.65909091 | 0.56363636 |
| 32 | 0.84444444 | 0.73484848 | 0.59393939 |
| 33 | 0.67777778 | 0.62121212 | 0.54848485 |
| 34 | 0.74444444 | 0.66666667 | 0.56666667 |
| 35 | 0.68888889 | 0.62878788 | 0.55151515 |
| 36 | 0.72222222 | 0.65151515 | 0.56060606 |
| 37 | 0.71111111 | 0.64393939 | 0.55757576 |
| 38 | 0.7 | 0.63636364 | 0.55454545 |
| 39 | 0.66666667 | 0.61363636 | 0.54545455 |
| 40 | 0.7 | 0.63636364 | 0.55454545 |
| 41 | 0.71111111 | 0.64393939 | 0.55757576 |
| 42 | 0.66666667 | 0.61363636 | 0.54545455 |
| 43 | 0.63333333 | 0.59090909 | 0.53636364 |
| 44 | 0.76666667 | 0.68181818 | 0.57272727 |
| 45 | 0.66666667 | 0.61363636 | 0.54545455 |
| 46 | 0.71111111 | 0.64393939 | 0.55757576 |
| 47 | 0.72222222 | 0.65151515 | 0.56060606 |
| 48 | 0.74444444 | 0.66666667 | 0.56666667 |
| 49 | 0.67777778 | 0.62121212 | 0.54848485 |
| 50 | 0.67777778 | 0.62121212 | 0.54848485 |
| 51 | 0.71111111 | 0.64393939 | 0.55757576 |
| 52 | 0.73333333 | 0.65909091 | 0.56363636 |
| 53 | 0.66666667 | 0.61363636 | 0.54545455 |
| 54 | 0.63333333 | 0.59090909 | 0.53636364 |
| 55 | 0.83333333 | 0.72727273 | 0.59090909 |
| 56 | 0.71111111 | 0.64393939 | 0.55757576 |
| 57 | 0.66666667 | 0.61363636 | 0.54545455 |
| 58 | 0.7 | 0.63636364 | 0.55454545 |
| 59 | 0.74444444 | 0.66666667 | 0.56666667 |
| 60 | 0.93333333 | 0.79545455 | 0.61818182 |
| 61 | 0.8 | 0.70454545 | 0.58181818 |
| 62 | 0.73333333 | 0.65909091 | 0.56363636 |
| 63 | 0.82222222 | 0.71969697 | 0.58787879 |
| 64 | 0.63333333 | 0.59090909 | 0.53636364 |
| 65 | 0.76666667 | 0.68181818 | 0.57272727 |
| 66 | 0.77777778 | 0.68939394 | 0.57575758 |
| 67 | 0.67777778 | 0.62121212 | 0.54848485 |
| 68 | 0.8 | 0.70454545 | 0.58181818 |
| 69 | 0.72222222 | 0.65151515 | 0.56060606 |
| 70 | 0.81111111 | 0.71212121 | 0.58484848 |
| 71 | 0.66666667 | 0.61363636 | 0.54545455 |
| 72 | 0.65555556 | 0.60606061 | 0.54242424 |
| 73 | 0.71111111 | 0.64393939 | 0.55757576 |
| 74 | 0.65555556 | 0.60606061 | 0.54242424 |
| 75 | 0.67777778 | 0.62121212 | 0.54848485 |
| 76 | 0.72222222 | 0.65151515 | 0.56060606 |
| 77 | 0.64444444 | 0.59848485 | 0.53939394 |
| 78 | 0.68888889 | 0.62878788 | 0.55151515 |
| 79 | 0.74444444 | 0.66666667 | 0.56666667 |
| 80 | 0.63333333 | 0.59090909 | 0.53636364 |
| 81 | 0.62222222 | 0.58333333 | 0.53333333 |
| 82 | 0.74444444 | 0.66666667 | 0.56666667 |
| 83 | 0.7 | 0.63636364 | 0.55454545 |
| 84 | 0.68888889 | 0.62878788 | 0.55151515 |
| 85 | 0.66666667 | 0.61363636 | 0.54545455 |
| 86 | 0.73333333 | 0.65909091 | 0.56363636 |
| 87 | 0.81111111 | 0.71212121 | 0.58484848 |
| 88 | 0.72222222 | 0.65151515 | 0.56060606 |
| 89 | 0.64444444 | 0.59848485 | 0.53939394 |
| 90 | 0.65555556 | 0.60606061 | 0.54242424 |
| 91 | 0.64444444 | 0.59848485 | 0.53939394 |
| 92 | 0.72222222 | 0.65151515 | 0.56060606 |
| 93 | 0.63333333 | 0.59090909 | 0.53636364 |
| 94 | 0.74444444 | 0.66666667 | 0.56666667 |
| 95 | 0.73333333 | 0.65909091 | 0.56363636 |
| 96 | 0.65555556 | 0.60606061 | 0.54242424 |
| 97 | 0.73333333 | 0.65909091 | 0.56363636 |
| 98 | 0.78888889 | 0.6969697 | 0.57878788 |
| 99 | 0.74444444 | 0.66666667 | 0.56666667 |
| 100 | 0.72222222 | 0.65151515 | 0.56060606 |

# Test runs for Metab grade 0 vs grade 3

Table S12: Test runs for Metab grade 0 vs grade 3 model.

| Test run | AUC | Sensitivity  Rate | Specificity Rate |
| --- | --- | --- | --- |
| 1 | 0.7047619 | 0.68695652 | 0.51483782 |
| 2 | 0.7047619 | 0.68695652 | 0.51483782 |
| 3 | 0.76825397 | 0.74492754 | 0.51943869 |
| 4 | 0.73650794 | 0.71594203 | 0.51713826 |
| 5 | 0.69206349 | 0.67536232 | 0.51391764 |
| 6 | 0.71746032 | 0.69855072 | 0.51575799 |
| 7 | 0.72063492 | 0.70144928 | 0.51598804 |
| 8 | 0.65079365 | 0.63768116 | 0.51092708 |
| 9 | 0.66984127 | 0.65507246 | 0.51230734 |
| 10 | 0.65714286 | 0.64347826 | 0.51138716 |
| 11 | 0.70793651 | 0.68985507 | 0.51506786 |
| 12 | 0.7047619 | 0.68695652 | 0.51483782 |
| 13 | 0.70793651 | 0.68985507 | 0.51506786 |
| 14 | 0.72380952 | 0.70434783 | 0.51621808 |
| 15 | 0.66666667 | 0.65217391 | 0.51207729 |
| 16 | 0.67619048 | 0.66086957 | 0.51276743 |
| 17 | 0.68571429 | 0.66956522 | 0.51345756 |
| 18 | 0.77142857 | 0.74782609 | 0.51966874 |
| 19 | 0.7015873 | 0.68405797 | 0.51460778 |
| 20 | 0.66984127 | 0.65507246 | 0.51230734 |
| 21 | 0.77460317 | 0.75072464 | 0.51989878 |
| 22 | 0.65714286 | 0.64347826 | 0.51138716 |
| 23 | 0.72380952 | 0.70434783 | 0.51621808 |
| 24 | 0.71111111 | 0.69275362 | 0.51529791 |
| 25 | 0.76507937 | 0.74202899 | 0.51920865 |
| 26 | 0.66031746 | 0.64637681 | 0.51161721 |
| 27 | 0.67619048 | 0.66086957 | 0.51276743 |
| 28 | 0.72063492 | 0.70144928 | 0.51598804 |
| 29 | 0.67936508 | 0.66376812 | 0.51299747 |
| 30 | 0.72380952 | 0.70434783 | 0.51621808 |
| 31 | 0.71428571 | 0.69565217 | 0.51552795 |
| 32 | 0.6952381 | 0.67826087 | 0.51414769 |
| 33 | 0.65079365 | 0.63768116 | 0.51092708 |
| 34 | 0.72380952 | 0.70434783 | 0.51621808 |
| 35 | 0.66984127 | 0.65507246 | 0.51230734 |
| 36 | 0.74603175 | 0.72463768 | 0.51782839 |
| 37 | 0.74920635 | 0.72753623 | 0.51805843 |
| 38 | 0.6952381 | 0.67826087 | 0.51414769 |
| 39 | 0.6952381 | 0.67826087 | 0.51414769 |
| 40 | 0.65714286 | 0.64347826 | 0.51138716 |
| 41 | 0.67619048 | 0.66086957 | 0.51276743 |
| 42 | 0.72380952 | 0.70434783 | 0.51621808 |
| 43 | 0.7047619 | 0.68695652 | 0.51483782 |
| 44 | 0.73333333 | 0.71304348 | 0.51690821 |
| 45 | 0.67936508 | 0.66376812 | 0.51299747 |
| 46 | 0.7015873 | 0.68405797 | 0.51460778 |
| 47 | 0.74603175 | 0.72463768 | 0.51782839 |
| 48 | 0.70793651 | 0.68985507 | 0.51506786 |
| 49 | 0.7015873 | 0.68405797 | 0.51460778 |
| 50 | 0.75555556 | 0.73333333 | 0.51851852 |
| 51 | 0.6952381 | 0.67826087 | 0.51414769 |
| 52 | 0.77142857 | 0.74782609 | 0.51966874 |
| 53 | 0.72380952 | 0.70434783 | 0.51621808 |
| 54 | 0.66666667 | 0.65217391 | 0.51207729 |
| 55 | 0.70793651 | 0.68985507 | 0.51506786 |
| 56 | 0.7047619 | 0.68695652 | 0.51483782 |
| 57 | 0.73968254 | 0.71884058 | 0.5173683 |
| 58 | 0.72380952 | 0.70434783 | 0.51621808 |
| 59 | 0.70793651 | 0.68985507 | 0.51506786 |
| 60 | 0.70793651 | 0.68985507 | 0.51506786 |
| 61 | 0.68888889 | 0.67246377 | 0.5136876 |
| 62 | 0.66031746 | 0.64637681 | 0.51161721 |
| 63 | 0.79365079 | 0.76811594 | 0.52127904 |
| 64 | 0.71428571 | 0.69565217 | 0.51552795 |
| 65 | 0.71746032 | 0.69855072 | 0.51575799 |
| 66 | 0.6952381 | 0.67826087 | 0.51414769 |
| 67 | 0.71746032 | 0.69855072 | 0.51575799 |
| 68 | 0.72063492 | 0.70144928 | 0.51598804 |
| 69 | 0.7015873 | 0.68405797 | 0.51460778 |
| 70 | 0.73333333 | 0.71304348 | 0.51690821 |
| 71 | 0.71111111 | 0.69275362 | 0.51529791 |
| 72 | 0.7047619 | 0.68695652 | 0.51483782 |
| 73 | 0.72380952 | 0.70434783 | 0.51621808 |
| 74 | 0.7015873 | 0.68405797 | 0.51460778 |
| 75 | 0.65079365 | 0.63768116 | 0.51092708 |
| 76 | 0.7047619 | 0.68695652 | 0.51483782 |
| 77 | 0.66349206 | 0.64927536 | 0.51184725 |
| 78 | 0.76190476 | 0.73913043 | 0.51897861 |
| 79 | 0.7015873 | 0.68405797 | 0.51460778 |
| 80 | 0.71428571 | 0.69565217 | 0.51552795 |
| 81 | 0.66349206 | 0.64927536 | 0.51184725 |
| 82 | 0.67301587 | 0.65797101 | 0.51253738 |
| 83 | 0.7047619 | 0.68695652 | 0.51483782 |
| 84 | 0.66349206 | 0.64927536 | 0.51184725 |
| 85 | 0.71111111 | 0.69275362 | 0.51529791 |
| 86 | 0.67936508 | 0.66376812 | 0.51299747 |
| 87 | 0.68253968 | 0.66666667 | 0.51322751 |
| 88 | 0.66031746 | 0.64637681 | 0.51161721 |
| 89 | 0.64761905 | 0.63478261 | 0.51069703 |
| 90 | 0.71428571 | 0.69565217 | 0.51552795 |
| 91 | 0.67301587 | 0.65797101 | 0.51253738 |
| 92 | 0.6031746 | 0.5942029 | 0.50747642 |
| 93 | 0.73333333 | 0.71304348 | 0.51690821 |
| 94 | 0.71428571 | 0.69565217 | 0.51552795 |
| 95 | 0.61587302 | 0.6057971 | 0.5083966 |
| 96 | 0.67619048 | 0.66086957 | 0.51276743 |
| 97 | 0.60952381 | 0.6 | 0.50793651 |
| 98 | 0.60634921 | 0.59710145 | 0.50770646 |
| 99 | 0.70793651 | 0.68985507 | 0.51506786 |
| 100 | 0.64126984 | 0.62898551 | 0.51023695 |

# Test runs for Metab grade 0 vs grade 4

Table S13: Test runs for Metab grade 0 vs grade 4 model.

| Test run | AUC | Sensitivity  Rate | Specificity Rate |
| --- | --- | --- | --- |
| 1 | 0.73333333 | 0.70491803 | 0.52727273 |
| 2 | 0.70909091 | 0.68548387 | 0.5202346 |
| 3 | 0.79393939 | 0.76075269 | 0.52844575 |
| 4 | 0.68484848 | 0.66397849 | 0.51788856 |
| 5 | 0.74848485 | 0.71584699 | 0.53025335 |
| 6 | 0.72424242 | 0.69892473 | 0.52170088 |
| 7 | 0.68484848 | 0.66120219 | 0.52280179 |
| 8 | 0.70606061 | 0.6827957 | 0.51994135 |
| 9 | 0.71212121 | 0.68817204 | 0.52052786 |
| 10 | 0.71212121 | 0.68817204 | 0.52052786 |
| 11 | 0.70606061 | 0.68032787 | 0.52637854 |
| 12 | 0.71515152 | 0.69086022 | 0.52082111 |
| 13 | 0.82424242 | 0.78763441 | 0.5313783 |
| 14 | 0.71212121 | 0.68306011 | 0.52309985 |
| 15 | 0.70606061 | 0.67759563 | 0.52369598 |
| 16 | 0.74545455 | 0.71774194 | 0.52375367 |
| 17 | 0.81515152 | 0.77956989 | 0.53049853 |
| 18 | 0.74545455 | 0.71311475 | 0.52876304 |
| 19 | 0.64545455 | 0.63661202 | 0.50730253 |
| 20 | 0.72121212 | 0.69125683 | 0.5266766 |
| 21 | 0.72727273 | 0.7016129 | 0.52199413 |
| 22 | 0.71515152 | 0.69086022 | 0.52082111 |
| 23 | 0.72424242 | 0.69892473 | 0.52170088 |
| 24 | 0.74848485 | 0.72043011 | 0.52404692 |
| 25 | 0.67575758 | 0.65591398 | 0.5170088 |
| 26 | 0.71212121 | 0.68817204 | 0.52052786 |
| 27 | 0.66363636 | 0.64516129 | 0.51583578 |
| 28 | 0.7969697 | 0.76344086 | 0.528739 |
| 29 | 0.67272727 | 0.65322581 | 0.51671554 |
| 30 | 0.69393939 | 0.66111111 | 0.53242424 |
| 31 | 0.66666667 | 0.64784946 | 0.51612903 |
| 32 | 0.81515152 | 0.77956989 | 0.53049853 |
| 33 | 0.73939394 | 0.71236559 | 0.52316716 |
| 34 | 0.65151515 | 0.6344086 | 0.51466276 |
| 35 | 0.7030303 | 0.68010753 | 0.51964809 |
| 36 | 0.71818182 | 0.69354839 | 0.52111437 |
| 37 | 0.84545455 | 0.80645161 | 0.53343109 |
| 38 | 0.72424242 | 0.69892473 | 0.52170088 |
| 39 | 0.72121212 | 0.69623656 | 0.52140762 |
| 40 | 0.77575758 | 0.74462366 | 0.52668622 |
| 41 | 0.68484848 | 0.65846995 | 0.52548435 |
| 42 | 0.65757576 | 0.63978495 | 0.51524927 |
| 43 | 0.65151515 | 0.6344086 | 0.51466276 |
| 44 | 0.76060606 | 0.7311828 | 0.52521994 |
| 45 | 0.67878788 | 0.65860215 | 0.51730205 |
| 46 | 0.74848485 | 0.72043011 | 0.52404692 |
| 47 | 0.57878788 | 0.56989247 | 0.50762463 |
| 48 | 0.71515152 | 0.69398907 | 0.51803279 |
| 49 | 0.76666667 | 0.73655914 | 0.52580645 |
| 50 | 0.65757576 | 0.63055556 | 0.52727273 |
| 51 | 0.71515152 | 0.69086022 | 0.52082111 |
| 52 | 0.73333333 | 0.70698925 | 0.52258065 |
| 53 | 0.69393939 | 0.67204301 | 0.51876833 |
| 54 | 0.70909091 | 0.68548387 | 0.5202346 |
| 55 | 0.65757576 | 0.63978495 | 0.51524927 |
| 56 | 0.72121212 | 0.69623656 | 0.52140762 |
| 57 | 0.75757576 | 0.72849462 | 0.52492669 |
| 58 | 0.75454545 | 0.72580645 | 0.52463343 |
| 59 | 0.7030303 | 0.68010753 | 0.51964809 |
| 60 | 0.70909091 | 0.68548387 | 0.5202346 |
| 61 | 0.70606061 | 0.6827957 | 0.51994135 |
| 62 | 0.74848485 | 0.72043011 | 0.52404692 |
| 63 | 0.7030303 | 0.68010753 | 0.51964809 |
| 64 | 0.64242424 | 0.62634409 | 0.51378299 |
| 65 | 0.70909091 | 0.68548387 | 0.5202346 |
| 66 | 0.7030303 | 0.67486339 | 0.52518629 |
| 67 | 0.65151515 | 0.6344086 | 0.51466276 |
| 68 | 0.68181818 | 0.66129032 | 0.51759531 |
| 69 | 0.82121212 | 0.78494624 | 0.53108504 |
| 70 | 0.77272727 | 0.74193548 | 0.52639296 |
| 71 | 0.67575758 | 0.65591398 | 0.5170088 |
| 72 | 0.77878788 | 0.74731183 | 0.52697947 |
| 73 | 0.72121212 | 0.69623656 | 0.52140762 |
| 74 | 0.71515152 | 0.69086022 | 0.52082111 |
| 75 | 0.81818182 | 0.78225806 | 0.53079179 |
| 76 | 0.65757576 | 0.63978495 | 0.51524927 |
| 77 | 0.72121212 | 0.69623656 | 0.52140762 |
| 78 | 0.67878788 | 0.6557377 | 0.51922504 |
| 79 | 0.64242424 | 0.62634409 | 0.51378299 |
| 80 | 0.68787879 | 0.66666667 | 0.51818182 |
| 81 | 0.75757576 | 0.72849462 | 0.52492669 |
| 82 | 0.68181818 | 0.66129032 | 0.51759531 |
| 83 | 0.67272727 | 0.65322581 | 0.51671554 |
| 84 | 0.78181818 | 0.75 | 0.52727273 |
| 85 | 0.6969697 | 0.66939891 | 0.52548435 |
| 86 | 0.69090909 | 0.66935484 | 0.51847507 |
| 87 | 0.7030303 | 0.68010753 | 0.51964809 |
| 88 | 0.70606061 | 0.67759563 | 0.52637854 |
| 89 | 0.76666667 | 0.73655914 | 0.52580645 |
| 90 | 0.7030303 | 0.68010753 | 0.51964809 |
| 91 | 0.65454545 | 0.63709677 | 0.51495601 |
| 92 | 0.61818182 | 0.60483871 | 0.51143695 |
| 93 | 0.66060606 | 0.64247312 | 0.51554252 |
| 94 | 0.77575758 | 0.74462366 | 0.52668622 |
| 95 | 0.62424242 | 0.61021505 | 0.51202346 |
| 96 | 0.58181818 | 0.57258065 | 0.50791789 |
| 97 | 0.59393939 | 0.58333333 | 0.50909091 |
| 98 | 0.57272727 | 0.56451613 | 0.50703812 |
| 99 | 0.57878788 | 0.56989247 | 0.50762463 |
| 100 | 0.5969697 | 0.58602151 | 0.50938416 |

# Test runs for Blood vs Infec

Table S14: Test runs for Blood vs Infec.

| Test run | AUC | Sensitivity  Rate | Specificity Rate |
| --- | --- | --- | --- |
| 1 | 0.78070175 | 0.75806452 | 0.51810979 |
| 2 | 0.79385965 | 0.77016129 | 0.51895869 |
| 3 | 0.66666667 | 0.65322581 | 0.51075269 |
| 4 | 0.69298246 | 0.67741935 | 0.51245048 |
| 5 | 0.72368421 | 0.70564516 | 0.51443124 |
| 6 | 0.66666667 | 0.65322581 | 0.51075269 |
| 7 | 0.6622807 | 0.64919355 | 0.51046972 |
| 8 | 0.67105263 | 0.65725806 | 0.51103565 |
| 9 | 0.70175439 | 0.68548387 | 0.51301641 |
| 10 | 0.70614035 | 0.68951613 | 0.51329938 |
| 11 | 0.70175439 | 0.68548387 | 0.51301641 |
| 12 | 0.71491228 | 0.69758065 | 0.51386531 |
| 13 | 0.71929825 | 0.7016129 | 0.51414827 |
| 14 | 0.6622807 | 0.64919355 | 0.51046972 |
| 15 | 0.69298246 | 0.67741935 | 0.51245048 |
| 16 | 0.71052632 | 0.69354839 | 0.51358234 |
| 17 | 0.69298246 | 0.67741935 | 0.51245048 |
| 18 | 0.6622807 | 0.64919355 | 0.51046972 |
| 19 | 0.72807018 | 0.70967742 | 0.5147142 |
| 20 | 0.72368421 | 0.70564516 | 0.51443124 |
| 21 | 0.66666667 | 0.65322581 | 0.51075269 |
| 22 | 0.75438596 | 0.73387097 | 0.516412 |
| 23 | 0.71052632 | 0.69354839 | 0.51358234 |
| 24 | 0.67105263 | 0.65725806 | 0.51103565 |
| 25 | 0.69298246 | 0.67741935 | 0.51245048 |
| 26 | 0.69736842 | 0.68145161 | 0.51273345 |
| 27 | 0.72368421 | 0.70564516 | 0.51443124 |
| 28 | 0.71929825 | 0.7016129 | 0.51414827 |
| 29 | 0.69298246 | 0.67741935 | 0.51245048 |
| 30 | 0.77631579 | 0.75403226 | 0.51782683 |
| 31 | 0.68421053 | 0.66935484 | 0.51188455 |
| 32 | 0.70175439 | 0.68548387 | 0.51301641 |
| 33 | 0.6754386 | 0.66129032 | 0.51131862 |
| 34 | 0.75877193 | 0.73790323 | 0.51669496 |
| 35 | 0.71052632 | 0.69354839 | 0.51358234 |
| 36 | 0.70614035 | 0.68951613 | 0.51329938 |
| 37 | 0.71491228 | 0.69758065 | 0.51386531 |
| 38 | 0.74561404 | 0.72580645 | 0.51584607 |
| 39 | 0.75877193 | 0.73790323 | 0.51669496 |
| 40 | 0.6622807 | 0.64919355 | 0.51046972 |
| 41 | 0.70175439 | 0.68548387 | 0.51301641 |
| 42 | 0.67982456 | 0.66532258 | 0.51160158 |
| 43 | 0.67105263 | 0.65725806 | 0.51103565 |
| 44 | 0.67105263 | 0.65725806 | 0.51103565 |
| 45 | 0.71491228 | 0.69758065 | 0.51386531 |
| 46 | 0.69736842 | 0.68145161 | 0.51273345 |
| 47 | 0.69298246 | 0.67741935 | 0.51245048 |
| 48 | 0.71491228 | 0.69758065 | 0.51386531 |
| 49 | 0.70614035 | 0.68951613 | 0.51329938 |
| 50 | 0.70175439 | 0.68548387 | 0.51301641 |
| 51 | 0.70175439 | 0.68548387 | 0.51301641 |
| 52 | 0.6622807 | 0.64919355 | 0.51046972 |
| 53 | 0.6622807 | 0.64919355 | 0.51046972 |
| 54 | 0.70175439 | 0.68548387 | 0.51301641 |
| 55 | 0.66666667 | 0.65322581 | 0.51075269 |
| 56 | 0.70175439 | 0.68548387 | 0.51301641 |
| 57 | 0.72368421 | 0.70564516 | 0.51443124 |
| 58 | 0.69736842 | 0.68145161 | 0.51273345 |
| 59 | 0.77192982 | 0.75 | 0.51754386 |
| 60 | 0.6622807 | 0.64919355 | 0.51046972 |
| 61 | 0.69298246 | 0.67741935 | 0.51245048 |
| 62 | 0.66666667 | 0.65322581 | 0.51075269 |
| 63 | 0.69298246 | 0.67741935 | 0.51245048 |
| 64 | 0.70175439 | 0.68548387 | 0.51301641 |
| 65 | 0.6754386 | 0.66129032 | 0.51131862 |
| 66 | 0.68421053 | 0.66935484 | 0.51188455 |
| 67 | 0.71929825 | 0.7016129 | 0.51414827 |
| 68 | 0.68859649 | 0.6733871 | 0.51216752 |
| 69 | 0.73684211 | 0.71774194 | 0.51528014 |
| 70 | 0.67982456 | 0.66532258 | 0.51160158 |
| 71 | 0.68859649 | 0.6733871 | 0.51216752 |
| 72 | 0.74122807 | 0.72177419 | 0.5155631 |
| 73 | 0.71491228 | 0.69758065 | 0.51386531 |
| 74 | 0.71929825 | 0.7016129 | 0.51414827 |
| 75 | 0.6622807 | 0.64919355 | 0.51046972 |
| 76 | 0.70175439 | 0.68548387 | 0.51301641 |
| 77 | 0.70175439 | 0.68548387 | 0.51301641 |
| 78 | 0.67105263 | 0.65725806 | 0.51103565 |
| 79 | 0.66666667 | 0.65322581 | 0.51075269 |
| 80 | 0.70175439 | 0.68548387 | 0.51301641 |
| 81 | 0.66666667 | 0.65322581 | 0.51075269 |
| 82 | 0.6622807 | 0.64919355 | 0.51046972 |
| 83 | 0.70614035 | 0.68951613 | 0.51329938 |
| 84 | 0.70614035 | 0.68951613 | 0.51329938 |
| 85 | 0.69298246 | 0.67741935 | 0.51245048 |
| 86 | 0.67982456 | 0.66532258 | 0.51160158 |
| 87 | 0.68421053 | 0.66935484 | 0.51188455 |
| 88 | 0.72807018 | 0.70967742 | 0.5147142 |
| 89 | 0.68421053 | 0.66935484 | 0.51188455 |
| 90 | 0.6622807 | 0.64919355 | 0.51046972 |
| 91 | 0.70175439 | 0.68548387 | 0.51301641 |
| 92 | 0.74122807 | 0.72177419 | 0.5155631 |
| 93 | 0.66666667 | 0.65322581 | 0.51075269 |
| 94 | 0.73245614 | 0.71370968 | 0.51499717 |
| 95 | 0.67105263 | 0.65725806 | 0.51103565 |
| 96 | 0.74561404 | 0.72580645 | 0.51584607 |
| 97 | 0.67982456 | 0.66532258 | 0.51160158 |
| 98 | 0.68421053 | 0.66935484 | 0.51188455 |
| 99 | 0.67105263 | 0.65725806 | 0.51103565 |
| 100 | 0.72368421 | 0.70564516 | 0.51443124 |

# Test runs for Blood vs Metab

Table S15: Test runs for Blood vs Metab.

| Test run | AUC | Sensitivity  Rate | Specificity Rate |
| --- | --- | --- | --- |
| 1 | 0.76724138 | 0.75409836 | 0.50876201 |
| 2 | 0.60344828 | 0.59836066 | 0.50339175 |
| 3 | 0.73275862 | 0.72131148 | 0.50763143 |
| 4 | 0.70689655 | 0.69672131 | 0.50678349 |
| 5 | 0.73275862 | 0.72131148 | 0.50763143 |
| 6 | 0.86206897 | 0.8442623 | 0.51187111 |
| 7 | 0.65517241 | 0.64754098 | 0.50508762 |
| 8 | 0.74137931 | 0.7295082 | 0.50791408 |
| 9 | 0.62931034 | 0.62711864 | 0.50496786 |
| 10 | 0.70689655 | 0.69672131 | 0.50678349 |
| 11 | 0.74137931 | 0.7295082 | 0.50791408 |
| 12 | 0.80172414 | 0.78688525 | 0.50989259 |
| 13 | 0.64655172 | 0.63934426 | 0.50480497 |
| 14 | 0.62068966 | 0.6147541 | 0.50395704 |
| 15 | 0.67241379 | 0.66393443 | 0.50565291 |
| 16 | 0.61206897 | 0.60655738 | 0.50367439 |
| 17 | 0.75 | 0.73770492 | 0.50819672 |
| 18 | 0.79310345 | 0.77868852 | 0.50960995 |
| 19 | 0.71551724 | 0.70491803 | 0.50706614 |
| 20 | 0.77586207 | 0.76229508 | 0.50904466 |
| 21 | 0.68965517 | 0.68032787 | 0.5062182 |
| 22 | 0.72413793 | 0.71311475 | 0.50734878 |
| 23 | 0.70689655 | 0.69672131 | 0.50678349 |
| 24 | 0.77586207 | 0.76229508 | 0.50904466 |
| 25 | 0.87931034 | 0.86065574 | 0.5124364 |
| 26 | 0.60344828 | 0.59836066 | 0.50339175 |
| 27 | 0.69827586 | 0.68852459 | 0.50650085 |
| 28 | 0.63793103 | 0.63114754 | 0.50452233 |
| 29 | 0.77586207 | 0.76229508 | 0.50904466 |
| 30 | 0.93103448 | 0.90983607 | 0.51413228 |
| 31 | 0.72413793 | 0.71311475 | 0.50734878 |
| 32 | 0.6637931 | 0.6557377 | 0.50537027 |
| 33 | 0.68965517 | 0.68032787 | 0.5062182 |
| 34 | 0.74137931 | 0.7295082 | 0.50791408 |
| 35 | 0.67241379 | 0.66393443 | 0.50565291 |
| 36 | 0.75 | 0.75 | 0.50028736 |
| 37 | 0.72413793 | 0.71311475 | 0.50734878 |
| 38 | 0.80172414 | 0.78688525 | 0.50989259 |
| 39 | 0.68965517 | 0.68032787 | 0.5062182 |
| 40 | 0.79310345 | 0.77868852 | 0.50960995 |
| 41 | 0.71551724 | 0.70491803 | 0.50706614 |
| 42 | 0.69827586 | 0.68852459 | 0.50650085 |
| 43 | 0.65517241 | 0.64754098 | 0.50508762 |
| 44 | 0.68103448 | 0.67213115 | 0.50593556 |
| 45 | 0.62931034 | 0.63333333 | 0.49741379 |
| 46 | 0.63793103 | 0.63114754 | 0.50452233 |
| 47 | 0.63793103 | 0.63114754 | 0.50452233 |
| 48 | 0.80172414 | 0.78688525 | 0.50989259 |
| 49 | 0.60344828 | 0.59836066 | 0.50339175 |
| 50 | 0.70689655 | 0.69672131 | 0.50678349 |
| 51 | 0.75862069 | 0.74590164 | 0.50847937 |
| 52 | 0.65517241 | 0.64754098 | 0.50508762 |
| 53 | 0.70689655 | 0.69672131 | 0.50678349 |
| 54 | 0.69827586 | 0.68852459 | 0.50650085 |
| 55 | 0.74137931 | 0.7295082 | 0.50791408 |
| 56 | 0.70689655 | 0.69672131 | 0.50678349 |
| 57 | 0.69827586 | 0.68852459 | 0.50650085 |
| 58 | 0.70689655 | 0.69672131 | 0.50678349 |
| 59 | 0.81896552 | 0.80327869 | 0.51045789 |
| 60 | 0.71551724 | 0.70491803 | 0.50706614 |
| 61 | 0.61206897 | 0.60655738 | 0.50367439 |
| 62 | 0.6637931 | 0.6557377 | 0.50537027 |
| 63 | 0.76724138 | 0.75409836 | 0.50876201 |
| 64 | 0.75862069 | 0.74590164 | 0.50847937 |
| 65 | 0.76724138 | 0.75409836 | 0.50876201 |
| 66 | 0.74137931 | 0.7295082 | 0.50791408 |
| 67 | 0.72413793 | 0.71311475 | 0.50734878 |
| 68 | 0.70689655 | 0.69672131 | 0.50678349 |
| 69 | 0.78448276 | 0.7704918 | 0.5093273 |
| 70 | 0.74137931 | 0.7295082 | 0.50791408 |
| 71 | 0.61206897 | 0.60655738 | 0.50367439 |
| 72 | 0.79310345 | 0.77868852 | 0.50960995 |
| 73 | 0.68965517 | 0.68032787 | 0.5062182 |
| 74 | 0.71551724 | 0.70491803 | 0.50706614 |
| 75 | 0.71551724 | 0.70491803 | 0.50706614 |
| 76 | 0.70689655 | 0.69672131 | 0.50678349 |
| 77 | 0.67241379 | 0.66393443 | 0.50565291 |
| 78 | 0.68965517 | 0.68032787 | 0.5062182 |
| 79 | 0.70689655 | 0.69672131 | 0.50678349 |
| 80 | 0.67241379 | 0.66393443 | 0.50565291 |
| 81 | 0.68103448 | 0.67213115 | 0.50593556 |
| 82 | 0.64655172 | 0.63934426 | 0.50480497 |
| 83 | 0.68965517 | 0.68032787 | 0.5062182 |
| 84 | 0.65517241 | 0.64754098 | 0.50508762 |
| 85 | 0.62068966 | 0.6147541 | 0.50395704 |
| 86 | 0.68103448 | 0.67213115 | 0.50593556 |
| 87 | 0.70689655 | 0.69672131 | 0.50678349 |
| 88 | 0.75 | 0.73770492 | 0.50819672 |
| 89 | 0.60344828 | 0.59836066 | 0.50339175 |
| 90 | 0.82758621 | 0.81147541 | 0.51074053 |
| 91 | 0.71551724 | 0.70491803 | 0.50706614 |
| 92 | 0.68965517 | 0.68032787 | 0.5062182 |
| 93 | 0.74137931 | 0.7295082 | 0.50791408 |
| 94 | 0.6637931 | 0.6557377 | 0.50537027 |
| 95 | 0.62068966 | 0.6147541 | 0.50395704 |
| 96 | 0.6637931 | 0.6557377 | 0.50537027 |
| 97 | 0.68103448 | 0.67213115 | 0.50593556 |
| 98 | 0.76724138 | 0.75409836 | 0.50876201 |
| 99 | 0.68965517 | 0.68032787 | 0.5062182 |
| 100 | 0.64655172 | 0.63934426 | 0.50480497 |

# Test runs for Infec vs Metab

Table S16: Test runs for Infec vs Metab.

| Test run | AUC | Sensitivity  Rate | Specificity Rate |
| --- | --- | --- | --- |
| 1 | 0.72380952 | 0.64596273 | 0.56811594 |
| 2 | 0.71428571 | 0.63975155 | 0.56521739 |
| 3 | 0.75238095 | 0.66459627 | 0.57681159 |
| 4 | 0.76190476 | 0.67080745 | 0.57971014 |
| 5 | 0.72380952 | 0.64596273 | 0.56811594 |
| 6 | 0.8 | 0.69565217 | 0.59130435 |
| 7 | 0.78095238 | 0.68322981 | 0.58550725 |
| 8 | 0.7047619 | 0.63354037 | 0.56231884 |
| 9 | 0.74285714 | 0.65838509 | 0.57391304 |
| 10 | 0.7047619 | 0.63354037 | 0.56231884 |
| 11 | 0.71428571 | 0.63975155 | 0.56521739 |
| 12 | 0.72380952 | 0.64596273 | 0.56811594 |
| 13 | 0.7047619 | 0.63354037 | 0.56231884 |
| 14 | 0.68571429 | 0.62111801 | 0.55652174 |
| 15 | 0.74285714 | 0.65838509 | 0.57391304 |
| 16 | 0.65714286 | 0.60248447 | 0.54782609 |
| 17 | 0.75238095 | 0.66459627 | 0.57681159 |
| 18 | 0.67619048 | 0.61490683 | 0.55362319 |
| 19 | 0.67619048 | 0.61490683 | 0.55362319 |
| 20 | 0.65714286 | 0.60248447 | 0.54782609 |
| 21 | 0.74285714 | 0.65838509 | 0.57391304 |
| 22 | 0.68571429 | 0.62111801 | 0.55652174 |
| 23 | 0.68571429 | 0.62111801 | 0.55652174 |
| 24 | 0.76190476 | 0.67080745 | 0.57971014 |
| 25 | 0.73333333 | 0.65217391 | 0.57101449 |
| 26 | 0.66666667 | 0.60869565 | 0.55072464 |
| 27 | 0.66666667 | 0.60869565 | 0.55072464 |
| 28 | 0.73333333 | 0.65217391 | 0.57101449 |
| 29 | 0.64761905 | 0.59627329 | 0.54492754 |
| 30 | 0.66666667 | 0.60869565 | 0.55072464 |
| 31 | 0.66666667 | 0.60869565 | 0.55072464 |
| 32 | 0.68571429 | 0.62111801 | 0.55652174 |
| 33 | 0.71428571 | 0.63975155 | 0.56521739 |
| 34 | 0.63809524 | 0.59006211 | 0.54202899 |
| 35 | 0.75238095 | 0.66459627 | 0.57681159 |
| 36 | 0.74285714 | 0.65838509 | 0.57391304 |
| 37 | 0.6952381 | 0.62732919 | 0.55942029 |
| 38 | 0.64761905 | 0.59627329 | 0.54492754 |
| 39 | 0.76190476 | 0.67080745 | 0.57971014 |
| 40 | 0.65714286 | 0.60248447 | 0.54782609 |
| 41 | 0.71428571 | 0.63975155 | 0.56521739 |
| 42 | 0.72380952 | 0.64596273 | 0.56811594 |
| 43 | 0.74285714 | 0.65838509 | 0.57391304 |
| 44 | 0.6952381 | 0.62732919 | 0.55942029 |
| 45 | 0.71428571 | 0.63975155 | 0.56521739 |
| 46 | 0.72380952 | 0.64596273 | 0.56811594 |
| 47 | 0.81904762 | 0.70807453 | 0.59710145 |
| 48 | 0.73333333 | 0.65217391 | 0.57101449 |
| 49 | 0.71428571 | 0.63975155 | 0.56521739 |
| 50 | 0.65714286 | 0.60248447 | 0.54782609 |
| 51 | 0.7047619 | 0.63354037 | 0.56231884 |
| 52 | 0.78095238 | 0.68322981 | 0.58550725 |
| 53 | 0.7047619 | 0.63354037 | 0.56231884 |
| 54 | 0.71428571 | 0.63975155 | 0.56521739 |
| 55 | 0.76190476 | 0.67080745 | 0.57971014 |
| 56 | 0.74285714 | 0.65838509 | 0.57391304 |
| 57 | 0.71428571 | 0.63975155 | 0.56521739 |
| 58 | 0.78095238 | 0.68322981 | 0.58550725 |
| 59 | 0.64761905 | 0.59627329 | 0.54492754 |
| 60 | 0.63809524 | 0.59006211 | 0.54202899 |
| 61 | 0.63809524 | 0.59006211 | 0.54202899 |
| 62 | 0.6952381 | 0.62732919 | 0.55942029 |
| 63 | 0.63809524 | 0.59006211 | 0.54202899 |
| 64 | 0.65714286 | 0.60248447 | 0.54782609 |
| 65 | 0.63809524 | 0.59006211 | 0.54202899 |
| 66 | 0.68571429 | 0.62111801 | 0.55652174 |
| 67 | 0.68571429 | 0.62111801 | 0.55652174 |
| 68 | 0.77142857 | 0.67701863 | 0.5826087 |
| 69 | 0.6952381 | 0.62732919 | 0.55942029 |
| 70 | 0.74285714 | 0.64935065 | 0.58787879 |
| 71 | 0.7047619 | 0.63354037 | 0.56231884 |
| 72 | 0.72380952 | 0.64596273 | 0.56811594 |
| 73 | 0.64761905 | 0.59627329 | 0.54492754 |
| 74 | 0.7047619 | 0.63354037 | 0.56231884 |
| 75 | 0.76190476 | 0.67080745 | 0.57971014 |
| 76 | 0.72380952 | 0.64596273 | 0.56811594 |
| 77 | 0.74285714 | 0.65838509 | 0.57391304 |
| 78 | 0.66666667 | 0.60869565 | 0.55072464 |
| 79 | 0.72380952 | 0.64596273 | 0.56811594 |
| 80 | 0.72380952 | 0.64596273 | 0.56811594 |
| 81 | 0.66666667 | 0.60869565 | 0.55072464 |
| 82 | 0.64761905 | 0.59627329 | 0.54492754 |
| 83 | 0.64761905 | 0.59627329 | 0.54492754 |
| 84 | 0.7047619 | 0.63354037 | 0.56231884 |
| 85 | 0.63809524 | 0.59006211 | 0.54202899 |
| 86 | 0.6952381 | 0.62732919 | 0.55942029 |
| 87 | 0.67619048 | 0.61490683 | 0.55362319 |
| 88 | 0.72380952 | 0.64596273 | 0.56811594 |
| 89 | 0.71428571 | 0.63975155 | 0.56521739 |
| 90 | 0.66666667 | 0.60869565 | 0.55072464 |
| 91 | 0.75238095 | 0.66459627 | 0.57681159 |
| 92 | 0.72380952 | 0.64596273 | 0.56811594 |
| 93 | 0.63809524 | 0.59006211 | 0.54202899 |
| 94 | 0.7047619 | 0.63354037 | 0.56231884 |
| 95 | 0.74285714 | 0.65838509 | 0.57391304 |
| 96 | 0.74285714 | 0.65838509 | 0.57391304 |
| 97 | 0.72380952 | 0.64596273 | 0.56811594 |
| 98 | 0.75238095 | 0.66459627 | 0.57681159 |
| 99 | 0.67619048 | 0.61490683 | 0.55362319 |
| 100 | 0.6952381 | 0.62732919 | 0.55942029 |

# Summary statistics for top five features: Blood grade 0 vs grade 3

Table S17: Top five feature summary for Blood grade 0 vs grade 3.

| Features | Lower Quartile | Mean | Upper Quartile |
| --- | --- | --- | --- |
| Ages 80 or above (Yrs) | No |  | Yes |
| Lactate Dehydrogenase (U/L) | 255.2 | 968.5 | 1000.8 |
| Alkaline Phosphatase (U/L) | 79 | 157.5 | 150 |
| Sodium (mMol/L) | 137.2 | 139.1 | 141 |
| Total Bilirubin (µMol/L) | 5.85 | 8.614 | 10.001 |

# Summary statistics for top five features: Blood grade 0 vs grade 4

Table S18: Top five feature summary for Blood grade 0 vs grade 4.

| Features | Lower Quartile | Mean | Upper Quartile |
| --- | --- | --- | --- |
| Male | No |  | Yes |
| Ages 65 to 69 (Yrs) | No |  | Yes |
| Platelets (10^9^/L) | 245 | 323.4 | 385 |
| Creatinine (µMol/L) | 63 | 76.52 | 89 |
| Haemoglobin (G/L) | 106 | 104.1 | 140 |

# Summary statistics for top five features: Blood grade 3 vs grade 4

Table S19: Top five feature summary for Blood grade 3 vs grade 4.

| Features | Lower Quartile | Mean | Upper Quartile |
| --- | --- | --- | --- |
| Female | No |  | Yes |
| Ages 60 to 64 (Yrs) | No |  | Yes |
| Haemoglobin (G/L) | 15.25 | 96 | 131.25 |
| Total Bilirubin (µMol/L) | 5.033 | 9.01 | 11.728 |
| Creatinine (µMol/L) | 64 | 79.38 | 92 |

# Summary statistics for top five features: Infec grade 0 vs grade 3

Table S20: Top five feature summary for Infec grade 0 vs grade 3.

| Features | Lower Quartile | Mean | Upper Quartile |
| --- | --- | --- | --- |
| Haemoglobin (G/L) | 15.3 | 97.62 | 134 |
| Total Bilirubin (µMol/L) | 6.84 | 9.233 | 11.97 |
| Leukocytes (10^9^/L) | 7 | 9.228 | 11.24 |
| Under 45 (Yrs) | No |  | Yes |
| Sodium (mMol/L) | 135 | 136.2 | 140.2 |

# Summary statistics for top five features: Infec grade 0 vs grade 4

Table S21: Top five feature summary for Infec grade 0 vs grade 4.

| Features | Lower Quartile | Mean | Upper Quartile |
| --- | --- | --- | --- |
| Haemoglobin (G/L) | 14.9 | 98.86 | 137.79 |
| Time since first diagnosis (Days) | 9 | 37.06 | 21.75 |
| Respiratory Medications | No |  | Yes |
| Total Bilirubin (µMol/L) | 5.939 | 8.415 | 9.426 |
| Leukocytes (10^9^/L) | 6.8 | 8.938 | 11.14 |

# Summary statistics for top three features: Infec grade 0 vs grade 5

Table S22: Top three feature summary for Infec grade 0 vs grade 5.

| Features | Lower Quartile | Mean | Upper Quartile |
| --- | --- | --- | --- |
| Haemoglobin (G/L) | 109 | 112 | 141 |
| Alkaline Phosphatase (U/L) | 84.5 | 184.9 | 241.5 |
| Creatinine (µMol/L) | 66 | 81.96 | 93 |

# Summary statistics for top five features: Infec grade 3 vs grade 4

Table S23: Top five feature summary for Infec grade 3 vs grade 4.

| Features | Lower Quartile | Mean | Upper Quartile |
| --- | --- | --- | --- |
| Platelets (10^9^/L) | 180.8 | 278.6 | 346.2 |
| Haemoglobin (G/L) | 13.12 | 45.09 | 43.35 |
| Ages 60 to 64 (Yrs) | No |  | Yes |
| Creatinine (µMol/L) | 63 | 73.19 | 80 |
| Female | No |  | Yes |

# Summary statistics for top five features: Metab grade 0 vs grade 3

Table S24: Top five feature summary for Metab grade 0 vs grade 3.

| Features | Lower Quartile | Mean | Upper Quartile |
| --- | --- | --- | --- |
| Total Bilirubin (µMol/L) | 5.001 | 8.99 | 8.85 |
| Albumin (G/L) | 33.5 | 38.11 | 41 |
| Ages 70 to 74 (Yrs) | No |  | Yes |
| Leukocytes (10^9^/L) | 7.575 | 9.913 | 11.725 |
| Aspartate Aminotransferase (U/L) | 18.05 | 33.68 | 45.5 |

# Summary statistics for top five features: Metab grade 0 vs grade 4

Table S25: Top five feature summary for Metab grade 0 vs grade 4.

| Features | Lower Quartile | Mean | Upper Quartile |
| --- | --- | --- | --- |
| Albumin (G/L) | 36.1 | 37.55 | 40.7 |
| Ages 60 to 64 (Yrs) | No |  | Yes |
| Sodium (mMol/L) | 134.8 | 135.4 | 140 |
| Creatinine (µMol/L) | 61.22 | 76.85 | 85.41 |
| Total Bilirubin (µMol/L) | 6.96 | 9.031 | 10.52 |

# Summary statistics for top five features: Blood vs Infec

Table S26: Top five feature summary for Blood vs Infec.

| Features | Lower Quartile | Mean | Upper Quartile |
| --- | --- | --- | --- |
| Haemoglobin (G/L) | 104 | 102.7 | 134 |
| Alkaline Phosphatase (U/L) | 88 | 132.9 | 153 |
| Leukocytes (10^9^/L) | 6.86 | 10.06 | 10.75 |
| Neutrophils (10^9^/L) | 4.22 | 6.085 | 6.618 |
| Sodium (mMol/L) | 136.4 | 138.8 | 141.4 |

# Summary statistics for top five features: Blood vs Metab

Table S27: Top five feature summary for Blood vs Metab.

| Features | Lower Quartile | Mean | Upper Quartile |
| --- | --- | --- | --- |
| Respiratory Medications | No |  | Yes |
| Platelets (10^9^/L) | 232.2 | 312.8 | 373 |
| Creatinine (µMol/L) | 65.81 | 74.78 | 87.75 |
| Ages 65 to 69 (Yrs) | No |  | Yes |
| Neutrophils (10^9^/L) | 4.473 | 5.721 | 6.045 |

# Summary statistics for top five features: Infec vs Metab

Table S28: Top five feature summary for Infec vs Metab.

| Features | Lower Quartile | Mean | Upper Quartile |
| --- | --- | --- | --- |
| Alkaline Phosphatase (U/L) | 86.75 | 132.56 | 125.25 |
| Haemoglobin (G/L) | 13.62 | 82.44 | 132.25 |
| Leukocytes (10^9^/L) | 7.04 | 11 | 12.18 |
| Total Bilirubin (µMol/L) | 5.001 | 7.776 | 8.662 |
| Female | No |  | Yes |
